# Marine-Derived Bioactive Compounds: A Promising Strategy for Ameliorating Skeletal Muscle Dysfunction in COPD

**DOI:** 10.3390/md23040158

**Published:** 2025-04-04

**Authors:** Meiling Jiang, Peijun Li, Xiaoyu Han, Linhong Jiang, Lihua Han, Qinglan He, Chen Yang, Zhichao Sun, Yingqi Wang, Yuanyuan Cao, Xiaodan Liu, Weibing Wu

**Affiliations:** 1 Department of Sports Rehabilitation, Shanghai University of Sport, Shanghai 200438, China; jml_1994@163.com (M.J.); hanxy78@163.com (X.H.); hanlihua889@163.com (L.H.); hql1538@163.com (Q.H.); yangchen99t@163.com (C.Y.); chao0409a@163.com (Z.S.); caoyuanyuan97@163.com (Y.C.); 2 School of Rehabilitation Science, Shanghai University of Traditional Chinese Medicine, Shanghai 201203, China; lpj0227@163.com (P.L.); 19821240232@163.com (L.J.); wangyingqii@163.com (Y.W.)

**Keywords:** marine-derived bioactive compounds, COPD, skeletal muscle dysfunction, polysaccharides, lipids, polyphenols, peptides, carotenoids

## Abstract

Chronic obstructive pulmonary disease (COPD) is frequently accompanied by skeletal muscle dysfunction, a critical and severe extrapulmonary complication. This dysfunction contributes to reduced exercise capacity, increased frequency of acute exacerbations, and elevated mortality, serving as an independent risk factor for poor prognosis in COPD patients. Owing to the unique physicochemical conditions of the marine environment, marine-derived bioactive compounds exhibit potent anti-inflammatory and antioxidant properties, demonstrating therapeutic potential for ameliorating COPD skeletal muscle dysfunction. This review summarizes marine-derived bioactive compounds with promising efficacy against skeletal muscle dysfunction in COPD, including polysaccharides, lipids, polyphenols, peptides, and carotenoids. The discussed compounds have shown bioactivities in promoting skeletal muscle health and suppressing muscle atrophy, thereby providing potential strategies for the prevention and treatment of COPD skeletal muscle dysfunction. These findings may expand the therapeutic strategies for managing COPD skeletal muscle dysfunction.

## 1. Introduction

Chronic obstructive pulmonary disease (COPD) is a heterogeneous pulmonary pathological condition characterized primarily by persistent respiratory symptoms and incompletely reversible airflow limitation resulting from structural abnormalities in the airways and/or alveoli [1]. The main clinical manifestations of COPD include dyspnea, cough, and sputum production. This condition is notable for its high incidence rates, diverse risk factors, significant mortality, and high disability rates. These characteristics impose a significant social and economic burden on countries worldwide and present a major challenge to global public health [2]. The incidence of COPD is increasing, particularly with advancing age, and is higher in males than in females. Consequently, COPD has become one of the most significant burdens on public health and the economy [3]. Skeletal muscle dysfunction is a common systemic manifestation associated with COPD. This dysfunction is primarily characterized by changes in muscle structure and function, which lead to decreased muscle strength and/or endurance [4], and these changes not only affect respiratory function but are also associated with poor exercise performance, increased fatigue, and a higher risk of fractures [5]. As a result, the management and treatment of skeletal muscle dysfunction have become an essential component of comprehensive health management in COPD patients.

Oceans cover more than 70% of the Earth’s surface and are the cradle of life on our planet. Marine flora and fauna represent around half of all global biodiversity and are the most valuable natural resources available to humanity. Marine ecosystems, owing to their extensive biodiversity such as extremes in temperature, pressure, salinity, and light levels, contain a substantial amount of protein and an abundance of essential amino acids, which offer potential health benefits and represent an important source of numerous bioactive compounds [6]. Marine invertebrates, fish, algae, and microorganisms are the primary groups known to produce bioactive compounds [7]. To date, over 30,000 natural products have been identified in marine environments, including unsaturated fatty acids, proteins, peptides, enzymes, polyphenols, polysaccharides, pigments, vitamins, and minerals [7,8]. 

The pathogenesis of skeletal muscle dysfunction in COPD has not yet been fully elucidated. However, inflammation and oxidative stress are acknowledged as critical factors contributing to the development of skeletal muscle dysfunction in COPD [9]. Marine species have been identified as sources of compounds with diverse therapeutic properties, including antioxidant, anti-inflammatory, antimicrobial, anticancer, neuroprotective, lipid-lowering, skin-protective, and sleep-enhancing effects [10]. Given these findings, the potential role of marine-derived bioactive compounds in addressing skeletal muscle dysfunction in COPD patients is increasingly being explored. This review seeks to provide a comprehensive summary of the potential of marine-derived bioactive compounds for skeletal muscle dysfunction in COPD.

## 2. COPD Skeletal Muscle Dysfunction

COPD is a multisystem disease characterized by heterogeneity and complexity. Patients with COPD not only exhibit respiratory symptoms due to airflow limitation but also present with a variety of extrapulmonary comorbidities [11,12]. These comorbidities are key factors leading to impaired activities of daily living in COPD patients and are closely associated with adverse prognosis and increased mortality [13]. Skeletal muscle dysfunction, characterized primarily by decreased muscle strength and endurance, is one of the main extrapulmonary comorbidities in many COPD patients, with an incidence rate of approximately 15% to 45% [14], and up to 63% in patients residing in nursing homes [15]. Skeletal muscle dysfunction affects muscle groups throughout the body, leading to reduced respiratory function and exercise capacity, which in turn cause physical inactivity. This dysfunction is an independent predictor of quality of life, cachexia, hospitalization rates, and survival in COPD patients [16,17]. Studies have shown that average muscle strength in COPD patients is reduced by 20% to 30%, and muscles in these patients are more prone to fatigue [18]. The decline in skeletal muscle endurance often precedes the decline in muscle strength, suggesting that endurance impairment may occur before strength impairment [19]. The severity of skeletal muscle dysfunction varies among different muscle groups. Inspiratory muscles are more severely affected than peripheral muscles, and lower limb muscles are more affected than upper limb muscles [20,21]. The manifestations of dysfunction in different muscle groups vary and may even be opposite, indicating that skeletal muscle dysfunction is influenced by local factors rather than being a complete systemic impairment [12]. The incidence and severity of skeletal muscle dysfunction are higher in COPD patients with cachexia and a reduced fat-free mass index (FFMI), but skeletal muscle dysfunction still exists in COPD patients with normal or even overweight body weight [22,23]. Improving skeletal muscle dysfunction can significantly enhance exercise capacity, improve activities of daily living, and increase survival rates in COPD patients. Therefore, the management of skeletal muscle dysfunction is an essential component of comprehensive management in COPD patients and has significant implications for the overall treatment and rehabilitation of COPD.

## 3. The Main Pathogenesis of Skeletal Muscle Dysfunction in COPD

### 3.1. Inflammation

COPD is recognized as a systemic chronic inflammatory disease, with the expression of inflammatory markers significantly correlating with disease severity [24]. A hypothesis has been proposed that skeletal muscle dysfunction in COPD patients may result from the "spillover" of inflammatory molecules from the lungs into the systemic circulation [13]. Existing studies have confirmed the presence of inflammation in the circulatory system and local skeletal muscles of COPD patients, primarily characterized by elevated expression of pro-inflammatory cytokines such as tumor necrosis factor-alpha (TNF-α), interleukin 6 (IL-6), and IL-8 [9]. Low-grade inflammation in the systemic circulation of COPD patients is substantially associated with decreased muscle strength, work capacity, and exercise endurance [25]. Inflammatory cytokines can activate the nuclear factor kappa-B (NF-κB) signaling pathway and other inflammatory factors, and interact with oxidative stress to activate the ubiquitin–proteasome system, thereby promoting the degradation of skeletal muscle proteins [26]. Furthermore, inflammation can also indirectly affect skeletal muscle function by influencing muscle metabolic types and mitochondrial function [27]. However, as research progresses, it has become increasingly evident that inflammation exhibits a dual role in maintaining muscle homeostasis. Chronic low-grade inflammation induces muscle catabolism through pleiotropic mechanisms mediated by inflammation. Conversely, acute inflammation caused by exercise or acute injury activates anabolic pathways and promotes muscle regeneration [9]. In summary, chronic low-grade inflammation emerges as one of the key pathogenic mechanisms underlying skeletal muscle dysfunction in COPD, affecting the structure and function of skeletal muscles through multiple pathways, ultimately contributing to the development of skeletal muscle dysfunction.

### 3.2. Oxidative Stress

Oxidative stress occurs when the balance between oxidants and antioxidants is disrupted in favor of oxidants, leading to the accumulation of reactive oxygen species (ROS). ROS accumulation disrupts redox balance and the mechanisms that regulate redox signaling, potentially causing molecular damage [28]. In the context of skeletal muscle, low concentrations of ROS play a role in regulating cellular signaling processes and are essential for normal force production [29]. However, under certain pathological conditions, these processes may be disrupted. Specifically, when ROS production exceeds the cellular antioxidant defense capacity, it results in either ROS accumulation or impairment of the antioxidant defense system. Consequently, this imbalance can trigger oxidative modifications of DNA, lipids, proteins, and carbohydrates, thereby impairing cellular function and ultimately causing damage to muscle structure and function [30]. Oxidative stress in both the systemic circulation and local skeletal muscles is considered a critical mechanism underlying the development of skeletal muscle dysfunction in COPD, a perspective that has gained consensus among researchers. Oxidative stress can interact with inflammation, mitochondrial dysfunction, and autophagy. These interactions disrupt the balance between muscle protein synthesis and degradation, thereby contributing to the progression of skeletal muscle dysfunction [31]. The role of oxidative stress in skeletal muscle within COPD is dual: on one hand, oxidative stress caused by the disease leads to structural and functional damage in skeletal muscle; on the other hand, oxidative stress induced by acute exercise activates the body’s antioxidant defense mechanisms [4,29]. This activation is an important basis for the improvement of oxidative stress in COPD skeletal muscle through long-term exercise. In summary, oxidative stress interacts with various pathological responses to participate in the development of skeletal muscle dysfunction in COPD.

### 3.3. Autophagy

Autophagy is a process of cellular self-degradation and serves as a crucial adaptive mechanism for cells to respond to changes in signaling and stress, playing a key role in cellular quality control and renewal [32]. While autophagy is an essential component for maintaining cellular health, its dysregulation can also contribute to disease progression. Multiple studies have found that muscle biopsies from patients with COPD show an increase in autophagy-related proteins and autophagosomes [33,34,35]. These proteins primarily include microtubule-associated protein 1A/1B-light chain 3 (LC3), LC3B-II, Bcl-2 interacting protein1 (Beclin 1), and sequestosome 1 (SQSTM1/p62). Concurrently, another investigation revealed elevated mRNA expression levels of autophagy-related proteins, such as UNC-51-like kinase 1 (ULK1), Beclin 1, LC3, p62, autophagy-related 7, whereas the formation rate of autophagosomes in muscle satellite cells was reduced [36]. Although direct data on autophagic flux in COPD skeletal muscle remain limited, it is evident that autophagy dysregulation occurs in the skeletal muscles of COPD patients experiencing muscle dysfunction.

### 3.4. Mitochondrial Dysfunction

Mitochondria serve as the primary site of ATP production within cells, participating in key metabolic pathways and functioning as essential organelles for maintaining cellular homeostasis and skeletal muscle health [37]. Mitochondria possess a stringent quality control system, which identifies and corrects mitochondrial dysfunction through processes such as mitophagy, fission, and fusion [38]. This system is vital for preserving skeletal muscle mass. In COPD patients with skeletal muscle dysfunction, the skeletal muscles exhibit mitochondrial abnormalities characterized by disruptions in mitochondrial biogenesis, fission, fusion, and autophagy.

#### 3.4.1. Decreased Mitochondrial Biogenesis

Mitochondrial biogenesis is the process of generating new mitochondria from existing ones, involving mtDNA replication, transcription, and translation, as well as mitochondrial fission, fusion, and quality control [39]. Peroxisome proliferator-activated receptor γ coactivator-1α (PGC-1α) is a major regulator of mitochondrial biogenesis. Through its role in activating various nuclear transcription factors, PGC-1α enhances the expression of mitochondrial transcription factor A (TFAM), thereby promoting mtDNA transcription and replication, which drives mitochondrial formation and function [40]. Previous studies have found that skeletal muscles from COPD patients exhibit reduced mitochondrial density and decreased expression of PGC-1α mRNA [41,42]. Additionally, these patients exhibit significant reductions in both the mtDNA/gDNA ratio and the expression of oxidative phosphorylation (OXPHOS) complexes [43]. The mtDNA/gDNA ratio is an important marker of mitochondrial content. Meanwhile, OXPHOS complexes refer to a series of protein complexes involved in oxidative phosphorylation. These complexes are located in the mitochondrial inner membrane and are responsible for generating ATP through the electron transport chain. Reduced expression of OXPHOS complexes may result in decreased mitochondrial energy production, which in turn may inhibit mitochondrial biogenesis. Therefore, these findings suggest that COPD patients with skeletal muscle dysfunction may experience a reduction in skeletal muscle mitochondrial biogenesis.

#### 3.4.2. Mitophagy

Autophagy maintains cellular metabolism and homeostasis by degrading surplus and impaired intracellular components [44]. Mitophagy refers to the process in which cells selectively engulf and degrade damaged or dysfunctional mitochondria, serving as a critical component of mitochondrial quality control. This process is essential for clearing defective mitochondria, maintaining cellular equilibrium, and participating in cell development, differentiation, defense against diseases, and therapeutic intervention [45]. Dysregulation of mitophagy has garnered increasing attention for its role in various pathologies. Mitophagy is finely regulated by multiple pathways and proteins, including PTEN-induced putative kinase 1 (PINK1)/Parkin RBR E3 ubiquitin protein ligase (Parkin), Bcl-2/adenovirus E1B interacting protein 3 (BNIP-3), and FUN14 domain-containing protein 1 (FUNDC1). While the coordinated regulation by these proteins ensures proper mitophagy function, any dysregulation of these factors may result in disease-associated phenotypes. Studies have found that skeletal muscles from COPD patients exhibit aberrant expression of mitophagy-related factors, including elevation of Bcl-2/adenovirus E1B interacting protein 3-like (BNIP-3L) protein and Parkin mRNA, and decreased expression of FUNDC1 protein and the LC3B-II/LC3B-I protein ratio [43]. Furthermore, another investigation revealed a significant reduction in Parkin protein abundance in muscle samples from COPD patients with skeletal muscle dysfunction [46]. Additional research employing Parkin knockout mice showed marked decreases in body weight, grip strength, and muscle mass, suggesting that insufficient Parkin-mediated mitophagy may represent a critical mechanism underlying skeletal muscle dysfunction in COPD [46]. Currently, the specific role of mitophagy in skeletal muscle dysfunction associated with COPD remains understudied. While existing studies have predominantly focused on alterations in the expression levels of autophagy-related proteins, the precise mechanisms by which mitophagy abnormalities contribute to the development of skeletal muscle dysfunction in COPD remain to be elucidated through further research.

#### 3.4.3. Mitochondrial Dynamics

**Figure 1:**
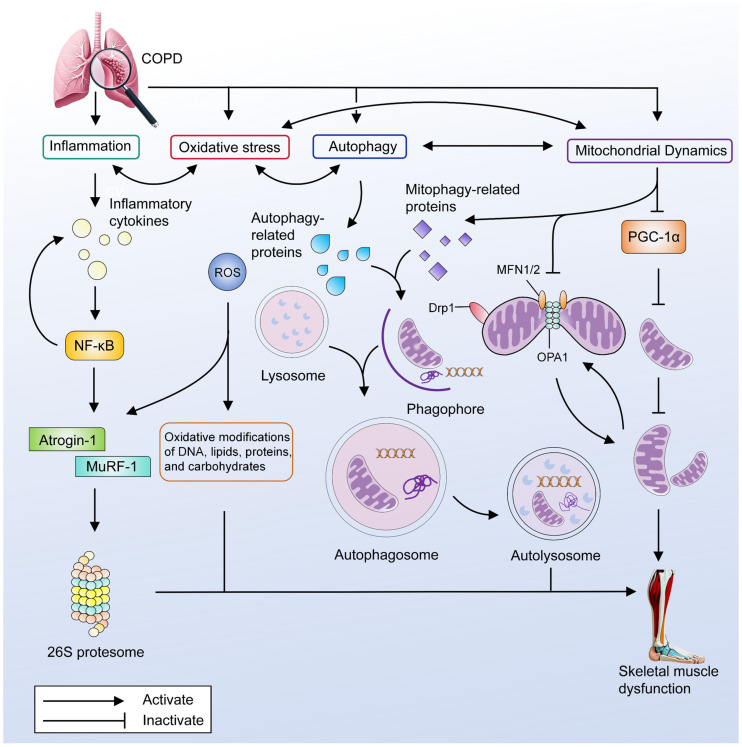
The primary pathogenesis of skeletal muscle dysfunction in COPD.

Mitochondrial dynamics encompasses changes in the morphology, number, and spatial distribution of mitochondria, including processes such as mitochondrial fission, fusion, transport, and quality control [47]. These dynamic processes are essential for maintaining normal mitochondrial function and cellular health. Among these, mitochondrial fission and fusion are particularly critical components of mitochondrial dynamics, playing pivotal roles in regulating the cell cycle, metabolism, and survival, while also being closely associated with various physiological and pathological conditions [48]. Mitochondrial fusion involves two primary mechanisms: outer membrane fusion mediated by mitofusin 1 (MFN1) and mitofusin 2 (MFN2), and inner membrane fusion facilitated by optic atrophy 1 protein (OPA1) [37]. Conversely, mitochondrial fission is primarily driven by a set of proteins, including dynamin-related protein 1 (Drp1), fission protein 1 (Fis1), mitochondrial fission factor (Mff), mitochondrial dynamics protein of 49 (MiD49), and MiD51 [37]. Fission serves to eliminate damaged or dysfunctional mitochondria, while fusion facilitates the exchange and homogenization of mitochondrial contents [49]. Mitochondrial fission and fusion are integral to preserving the structural integrity and functional efficiency of skeletal muscle mitochondria. A previous study has demonstrated that in the vastus lateralis muscle of COPD patients without confirmed skeletal muscle dysfunction, the expression of Drp1, a key regulator of mitochondrial fission, is decreased [43]. In spite of that, the expression of Drp1 was increased in the quadriceps of COPD rats and in C2C12 cells stimulated by cigarette smoke extract [50]. Currently, research on mitochondrial fission and fusion in skeletal muscles of COPD patients with skeletal muscle dysfunction is still in infancy. Therefore, while interpreting existing data cautiously is advisable, the observed abnormalities in mitochondrial dynamics within the skeletal muscles of COPD patients with skeletal muscle dysfunction undeniably call for further investigation. The primary pathogenesis of skeletal muscle dysfunction in COPD is summarized in Figure 1.

## 4. Potential of Marine-Derived Bioactive Compounds on COPD Skeletal Muscle Dysfunction

The ocean’s ecosystem, rich in biodiversity and chemical complexity, harbors approximately 90% of the global biomass, which contributes to a wealth of bioactive compounds with potential applications in health and medicine. Numerous natural products have been identified in marine environments, including lipids, peptides, polyphenols, polysaccharides, pigments, enzymes, vitamins, and minerals [7,8]. Among them, polysaccharides, lipids, polyphenols, peptides, and carotenoids demonstrate potential bioactivity for preventing skeletal muscle dysfunction in COPD, as summarized in Table 1.

### 4.1. Polysaccharides

Polysaccharides are polymeric carbohydrates composed of long chains of monosaccharide units linked together by glycosidic bonds, typically with a molecular weight of at least 10 monosaccharide units [83]. Marine polysaccharides can be categorized by source into marine algae-derived polysaccharides, marine animal-derived polysaccharides, and polysaccharides metabolized by marine microorganisms. Among these, fucoidan from algae and chitosan from marine animals demonstrate therapeutic potential for COPD skeletal muscle dysfunction. 

#### 4.1.1. Fucoidan

**Figure 2:**
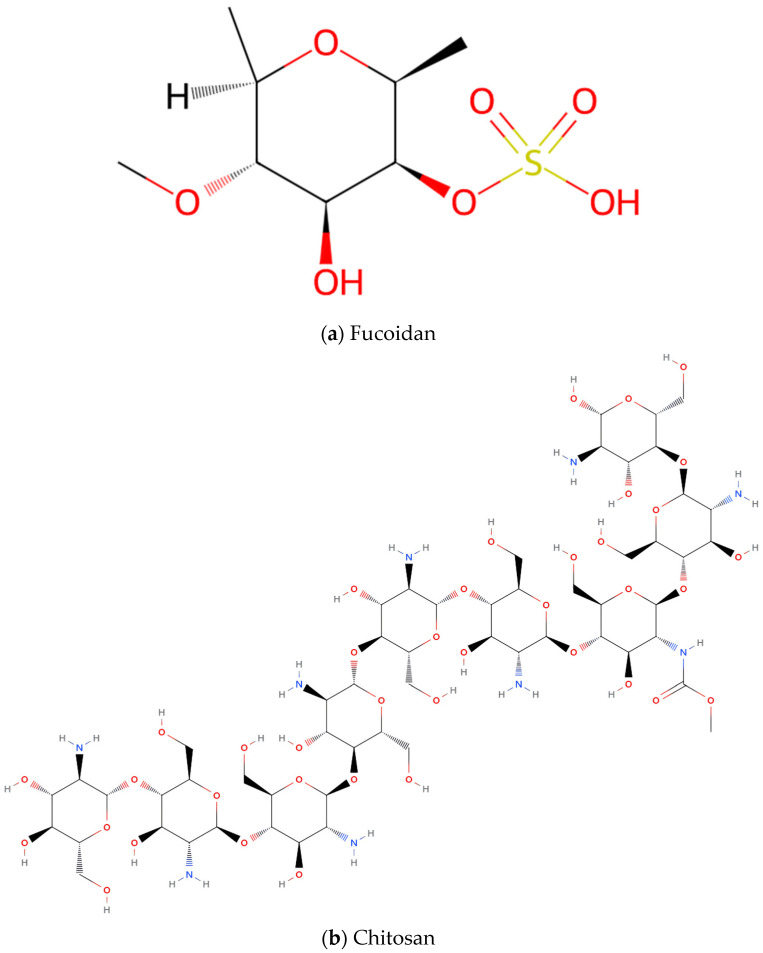
Chemical structures of polysaccharides with potentially therapeutic effects on COPD skeletal muscle dysfunction: (**a**) Fucoidan, (**b**) Chitosan.

Fucoidan ([(2*S*,3*S*,4*S*,5*S*,6*R*)-4-hydroxy-5-methoxy-2,6-dimethyloxan-3-yl] hydrogen sulfate; Figure 2a), a sulfated heteropolysaccharide predominantly extracted from brown algae with the chemical formula (C_6_H_10_O_7_S)n, is characterized by its high content of L-fucose residues and sulfate ester groups [84]. Accumulating evidence from preclinical studies highlights its beneficial effects on muscle endurance, mass, and fatigue resistance in rodent models [51,52,53,54]. Administration of fucoidan at doses of 310 and 620 mg/kg/day for 21 days significantly improved grip strength and swimming endurance in mice, while reducing post-exercise fatigue biomarkers including blood lactate, ammonia, and blood urea nitrogen (BUN) in a dose-dependent manner [51]. Histopathological examinations confirmed no adverse effects on hepatic, skeletal muscle, cardiac, renal, or pulmonary tissues. Fucoidan supplementation (400 mg/kg/day) enhanced skeletal muscle performance through increased muscle fiber cross-sectional area (CSA) and contractile force, although it did not significantly improve exercise training adaptations [53]. Prolonged fucoidan supplementation (400 mg/kg/day) for 10 weeks increased muscle mass and running distance, accompanied by upregulation of mRNA levels for mitochondrial biogenesis and oxidative metabolism regulators, including cytochrome c oxidase subunit 4 (COX4), myosin heavy chain 1 (MYH1), PGC-1α, PPAR-γ, and insulin-like growth factor 1 (IGF-1) [52]. Fucoidan has also been explored in preliminary human clinical trials. A 12-day human trial (1 g/day) demonstrated no significant enhancement in exercise performance. However, fucoidan supplementation attenuated post-exercise inflammatory responses, suggesting potential benefits in accelerating recovery following high-intensity exercise [54]. Fucoidan’s combined effects on muscle endurance, hypertrophy, and fatigue resistance, together with its antioxidative properties, position it as a promising therapeutic candidate for COPD skeletal muscle dysfunction (Table 2). These benefits may counteract muscle atrophy and exercise intolerance commonly observed in COPD patients. Further clinical studies in human populations are needed to validate optimal dosing strategies and long-term safety.

#### 4.1.2. Chitosan

Chitosan, with the chemical formula (C_6_H_11_NO_4_)_n_, is a linear polysaccharide composed of D-glucosamine and N-acetyl-D-glucosamine units linked by *β*-(1,4)-glycosidic bonds (Figure 2b) [85]. While it is naturally derived from marine crustacean shells or fungal cell walls, its poor water solubility limits its direct application in food and biomedical industries [86]. To address this, chitosan is often enzymatically or chemically hydrolyzed into water-soluble chitosan oligosaccharides (COS), which exhibit enhanced mitochondrial function modulation, antioxidant activity, and anti-fatigue properties [55,56,57]. Preclinical studies in mice revealed that 2 weeks of COS supplementation significantly improved fatigue resistance by increasing mitochondrial membrane potential, mitochondrial quantity, and SOD activity while reducing malondialdehyde (MDA) levels [57]. Similarly, a 6-week dietary intervention with COS (0.5% (*w*/*w*)) in Sprague-Dawley (SD) rats enhanced skeletal muscle mitochondrial content and exercise endurance without hepatotoxicity [56]. In vitro experiments further demonstrated that COS treatment increased mitochondrial density and upregulated mitochondrial electron transport chain (ETC) component proteins in C2C12 myotubes, accompanied by elevated mRNA expression of mitochondrial biogenesis regulators (PGC-1α, nuclear respiratory factor 1 (Nrf1), and TFAM), mediated through Sirtuin 1 (SIRT1) and AMP-activated protein kinase (AMPK) signaling pathways. COS also promoted glucose uptake in C2C12 myotubes, suggesting metabolic benefits [55]. However, contradictory evidence emerged from a 6-week rat study reporting reduced activity of skeletal muscle mitochondrial respiratory chain complexes, indicating potential mitochondrial toxicity with prolonged COS use [58]. In summary, COS shows therapeutic potential for treating COPD skeletal muscle dysfunction, due to its anti-fatigue effects and ability to enhance mitochondrial function (Table 2). However, its effects vary depending on usage duration. Short-term interventions demonstrate clear benefits, whereas prolonged exposure may carry toxicity risks. These contrasting outcomes emphasize the need for further research to determine optimal dosing strategies and assess long-term safety.

**Table 2:** Potential Ameliorative Effects of Polysaccharides in COPD Skeletal Muscle Dysfunction.

Ref.	Object, Sample Size	Intervention	Origin	Administration Route	Dose	Duration	Primary Results
Yang, C. (2024) [52]	C57BL/6J mice (16/16)	Fucoidan	*Undaria pinnatifida*	Incorporate a non-caloric sweetener for oral use	400 mg/kg/d	10 weeks	Running Distance ↑; Muscle mass ↑; COX4 mRNA ↑; MYH1 mRNA ↑; PGC-1α mRNA ↑; PPAR-γ mRNA ↑; IGF-1 mRNA ↑
McFadden, B. A. (2023) [54]	Healthy adult (8/8)	Fucoidan	*Undaria pinnatifida*	Oral	1 g/day	12 days	IL-6 and IL-10 concentrations 30 min post-exercise ↑
McBean, S.E. (2021) [53]	C57BL/6J mice (8/10)	Fucoidan	*Undaria pinnatifida and Fucus vesiculosus*	Oral gavage	400 mg/kg/d	4 weeks	CSA of EDL and soleus fibers ↑; TA force ↑; MHC-2x of gastrocnemius ↑
Chen, Y.M. (2014) [51]	ICR mice (8/8)	Fucoidan	*Laminaria japonica*	Oral gavage	310 mg/kg/d 620 mg/kg/d	21 days	Forelimb Grip Strength ↑; Weight-loaded swimming test time ↑; Serum lactate levels after acute exercise challenge ↓; Serum ammonia levels after acute exercise challenge ↓; Serum glucose levels after acute exercise challenge ↑; Serum levels of total protein ↑; Serum levels of blood urea nitrogen ↓; Serum levels of triacylglycerol ↓
Ha, B.G. (2016) [55]	C2C12 myotube	COS	Commercial sources	-	2 mg/mL	1 h	Glucose uptake ↑
Teodoro, J.S (2016) [58]	Wistar rat (ND)	COS	Commercial sources	Feed the water with 0.5% COS	0.5% (*w*/*w*)	6 weeks	SDH activity ↓; COX activity ↓; ATPSynthase activity ↓
Jeong, H.W. (2012) [56]	Sprague-Dawley rat (12/12)/C2C12 myotube	COS	Commercial sources	Feed the chow with 0.5% (*w*/*w*) COS/-	0.5% (*w*/*w*)/10 μg/mL; 100 μg/mL; 500 μg/mL	6 weeks/ 24 h or 12 h	Mitochondrial content of soleus ↑; PGC1α mRNA ↑; Nrf1 mRNA ↑; CPT1b mRNA ↑; TFAM mRNA ↑/Mitochondrial content ↑; NDFUA9 protein ↑; SDHA protein ↑; UQCRC2 protein ↑; COX1 protein ↑; ATP5a protein ↑
Cho, S.Y. (2010) [57]	BALB/c mice	COS lactate	Commercial sources	Oral	500 mg/kg	2 weeks	Immobility time in a forced swimming test ↑; Cortisol ↓; LDH ↓; SOD activity ↑; MDA ↓ Mitochondrial mass ↑; Membrane potential ↑; PGC-1α ↑; Cyt C ↑

ATP5a, ATP synthase mitochondrial F1 complex alpha subunit 1; Akt, protein Kinase B; COX, cytochrome c oxidase subunit; CSA, cross-sectional area; Cyt C, cytochrome c; COS, chitosan oligosaccharides; EDL, Extensor digitorum longus; GPR84, G protein-coupled receptor 84; IGF-1, insulin-like growth factor 1; L-6, interleukin 6; IL-8, interleukin 8; LDH, lactate dehydrogenase; MYH1, myosin heavy chain 1; MHC, myosin heavy chain; MDA, malondialdehyde; NDUFA9, NADH dehydrogenase 1 alpha subcomplex 9; ND, not described; PGC-1α, peroxisome proliferator-activated receptor gamma coactivator 1α; PPAR-γ, peroxisome proliferator-activated receptor gamma; PI3K, phosphoinositide 3-kinases; SOD, superoxide dismutase; SDH, succinate dehydrogenase; SDHA, succinate dehydrogenase complex subunit A; TA, tibialis anterior; UQCRC2, ubiquinol–cytochrome c reductase core protein.

### 4.2. Lipids

Marine functional lipids, derived from a variety of marine organisms such as marine fungi, microalgae, fish, and large marine animals, hold significant value in production and scientific research. Owing to the unique marine environment, these lipids exhibit distinct chemical structures, mechanisms of action, and functional characteristics compared to terrestrial biological lipids. The biological activity of marine lipids toward human health has been demonstrated in diverse fields. Specifically, long-chain omega-3 polyunsaturated fatty acids (n-3 LC-PUFAs) and sterols are currently under investigation for their therapeutic effects on skeletal muscle dysfunction in COPD patients.

#### 4.2.1. Long-Chain Omega-3 Polyunsaturated Fatty Acids

**Figure 3:**
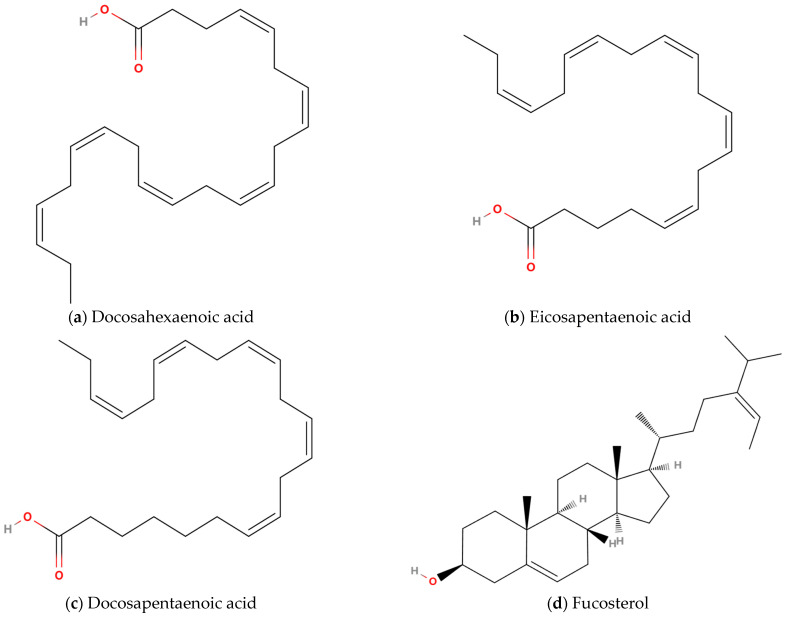
Chemical structures of lipids with potentially therapeutic effects on COPD skeletal muscle dysfunction: (**a**) Docosahexaenoic acid, (**b**) Eicosapentaenoic acid, (**c**) Docosapentaenoic acid, (**d**) Fucosterol.

n-3 LC-PUFAs, comprising docosahexaenoic acid (DHA, (4*Z*,7*Z*,10*Z*,13*Z*,16*Z*,19*Z*)-docosa-4,7,10,13,16,19-hexaenoic acid; Figure 3a), eicosapentaenoic acid (EPA, (5*Z*,8*Z*,11*Z*,14*Z*,17*Z*)-icosa-5,8,11,14,17-pentaenoic acid; Figure 3b), and docosapentaenoic acid (DPA, (7*Z*,10*Z*,13*Z*,16*Z*,19*Z*)-docosa-7,10,13,16,19-pentaenoic acid; Figure 3c), are distinguished by their anti-inflammatory and inflammation-resolving pharmacological activities [87]. DPA, an intermediate metabolite derived from EPA, is found in trace quantities within marine fish [88]. Mammalian organisms lack the enzymatic machinery necessary for the synthesis of EPA and DHA, but they can be endogenously biosynthesized through sequential elongation and desaturation of α-linolenic acid (ALA, 18:3n-3), a plant-originated precursor [89]. The primary dietary sources of n-3 LC-PUFAs are fish oil extracted from the adipose tissues of fatty fish, the liver of white lean fish, and the blubber of marine mammals [90]. In nutraceutical and therapeutic contexts, n-3 LC-PUFAs are typically formulated as EPA-DHA conjugates. Although these molecules exhibit overlapping biofunctional profiles, data suggest divergent modulatory roles in pro-inflammatory signaling cascades and anti-inflammatory countermeasures, a phenomenon likely attributable to structural variance. Specifically, DHA (C_22_H_32_O_2_, 22:6n-3) is characterized by a 22-carbon backbone with six conjugated double bonds, whereas EPA (C_20_H_30_O_2_, 20:5n-3) possesses a 20-carbon chain featuring five non-methylene-interrupted double bonds. The heightened unsaturation index of DHA enhances its capacity to perturb the biophysical properties of phospholipid bilayers, notably augmenting membrane microviscosity reduction and transmembrane proton conductance efficiency in planar lipid bilayer models, a trait surpassing that observed in EPA [91]. In COPD patients with skeletal muscle dysfunction, plasma levels of DHA are reduced [92]. Supplementation with n-3 LC-PUFAs has been extensively employed in chronic wasting diseases to mitigate muscle loss [93], and a meta-analysis has demonstrated their positive impact on whole-body muscle mass and strength [94]. Therefore, the potential effectiveness of n-3 LC-PUFAs in addressing skeletal muscle dysfunction in COPD has garnered significant research interest.

Cross-sectional studies have revealed that COPD patients aged 40 years or older who consistently consumed 0.5 g of n-3 LC-PUFAs daily for 6 months exhibited increased 6-min walk test distances and quality of life, along with reduced disease exacerbation frequency, bronchodilator use, and serum C-reactive protein (CRP) levels [61]. Furthermore, an analysis of dietary data from 250 stable COPD patients indicated that higher intake of n-3 LC-PUFAs was associated with lower serum TNF-α concentrations [62]. In a subgroup of stable COPD patients without skeletal muscle dysfunction, daily supplementation with 1.68 g EPA and 522 mg DHA for 10 weeks significantly enhanced lean soft tissue content, although no substantial improvements were observed in respiratory muscles, skeletal muscles, brain health, or physical activity capacity [63]. 

Evidence-based medicine has confirmed that pulmonary rehabilitation training is an effective intervention for both stable and acute exacerbated COPD patients [95]. However, whether combining n-3 LC-PUFAs with pulmonary rehabilitation training would yield better outcomes remains a topic of growing interest among scholars. Studies have demonstrated that irrespective of n-3 PUFAs supplementation, 8 weeks of exercise rehabilitation training was able to enhance various physiological measures in patients, including fat-free mass, peak load, peak oxygen uptake, peak carbon dioxide output, and quadriceps muscle strength [96]. However, patients supplemented with n-3 LC-PUFAs exhibited greater improvements in peak load, fat mass (FM), and exercise duration. Notably, no significant changes were observed in serum CRP, IL-6, and TNF-α expression levels across both groups. These findings suggest that supplementation with n-3 LC-PUFAs may have a beneficial impact on the exercise capacity of COPD patients, primarily by improving muscle endurance rather than muscle strength. Furthermore, a 12-week intervention involving nutritional supplementation and pulmonary rehabilitation training was conducted among COPD patients with cachexia, and yielded favorable tolerability results [97]. The intervention demonstrated its efficacy in increasing FM, reducing fatigue and dyspnea associated with the 6-min walk test, and showing a trend toward lowering inflammatory cytokine levels. Additionally, a 4-month high-intensity pulmonary rehabilitation training program, combined with nutrient supplementation rich in n-3 LC-PUFAs was implemented among COPD patients with skeletal muscle dysfunction [92]. The results demonstrated that the n-3 LC-PUFAs supplements showed an advantage in improving respiratory muscle strength. Existing studies have demonstrated the beneficial effects and safety of n-3 LC-PUFAs on skeletal muscle dysfunction in COPD populations (Table 3). However, it is currently unclear whether the amelioration of skeletal muscle dysfunction in COPD that is attributed to n-3 LC-PUFAs is primarily mediated through reductions in inflammatory responses.

#### 4.2.2. Sterols

Marine sterols, a class of sterol compounds synthesized by marine organisms, represent the most abundant natural organic compounds in oceanic ecosystems, primarily derived from marine algae. Fucosterol (FST, (3S,8S,9S,10R,13R,14S,17R)-10,13-dimethyl-17-[(E,2R)-5-propan-2-ylhept-5-en-2-yl]-2,3,4,7,8,9,11,12,14,15,16,17-dodecahydro-1H-cyclopenta[a]phenanthren-3-ol, C_29_H_48_O; Figure 3d), predominantly present in brown algae, constitutes 83–97% of total algal sterol content [98]. Fucosterol is a 3β-sterol derived from stigmastan-3β-ol, containing double bonds at positions 5 and 24(28) (with the 24(28) double bond adopting an E-configuration), a 3β-hydroxyl group at the C3 position, and a 24-ethylidene group on the C24 side chain [99]. These structural features collectively confer potent antioxidant and anti-inflammatory properties on FST. The antioxidant activity of FST was first experimentally validated by Lee et al. [64], while Jung et al. [100] provided the first experimental evidence of its anti-inflammatory effects. Subsequent studies have corroborated these bioactivities and elucidated the underlying molecular mechanisms: FST exerts antioxidant effects primarily through activation of the nuclear factor erythroid 2-related factor 2/heme oxygenase-1 (Nrf2/HO-1) signaling pathway, whereas its anti-inflammatory action involves inhibition of NF-κB and mitogen-activated protein kinase (MAPK) signaling pathway phosphorylation [101,102,103]. Notably, FST demonstrates protective effects against inflammation-induced pulmonary tissue damage. Experimental evidence indicates its capacity to attenuate lipopolysaccharide-induced acute lung injury and pulmonary macrophage inflammation in murine models through suppression of pro-inflammatory cytokines [104], as well as to ameliorate air pollutant-triggered pulmonary inflammation and oxidative stress [105]. The combined antioxidant and anti-inflammatory profile and lung-protective properties of FST position it as a promising therapeutic candidate for mitigating COPD skeletal muscle dysfunction (Table 3).

**Table 3:** Potential Ameliorative Effects of Lipids in COPD Skeletal Muscle Dysfunction.

Ref.	Object, Sample Size	Intervention	Origin	Administration Route	Dose	Duration	Primary Results
Engelen M (2024) [63]	Patients with COPD of grade II–IV (16/16)	n-3 LC-PUFAs	ND	Oral	1.68 g EPA + 522 mg DHA/d	10 weeks	Lean soft tissue ↑
Fekete M (2022) [61]	Patients with COPD (19/381)	n-3 LC-PUFAs	ND	Oral	0.5 g/day	6 months	BMI ↑; CAT ↑; Inhaled short-acting bronchodilators use ↓; Number of exacerbations in the previous half year ↓; 6MWD ↑
Ogasawara T (2018) [106]	Patients with COPD of exacerbation (24/21)	EPA + pulmonary rehabilitation	ND	Oral	1 g/day	12 days	Insignificant increase in LBMI and SMI
Calder, P C (2018) [97]	COPD patients with cachexia (20/19)	Nutrients rich in n-3 LC-PUFAs, and 25-hydroxy-vitamin D3 + pulmonary rehabilitation	ND	Oral	200 mL/unit, 2 units/d; ~230 kcal, 10 g whey protein concentrate, 2.0 g DHA + EPA, and 10 μg 25-hydroxy-vitamin D3 per unit	12 weeks	FM ↑; Dyspnea ↓; Anti-fatigue ↑
van de Bool C (2017) [92]	COPD patients with low muscle mass (38/42)	Nutrients riche leucine, n-3 LC-PUFAs, and vitamin D	ND	Oral	125 mL/unit, 2~3 units/d, 187.5 kCal in a distribution of 20 energy percent protein, 60 energy percent carbohydrate, and 20 energy percent fat, and is enriched with leucine, n-3 PUFAs, and vitamin D per unit	4 months	Inspiratory muscle strength ↑

BMI, body mass index; CAT, COPD Assessment Test; DHA, docosahexaenoic acid; EPA, eicosapentaenoic acid; LBMI, lean body mass index; ND, not described; SMI, skeletal muscle mass index; 6MWD, 6-min walking distance.

### 4.3. Polyphenols

Marine polyphenols are bioactive compounds widely distributed in various marine organisms. Their molecular structure contains multiple hydroxyl (-OH) groups, which confer antioxidant and anti-inflammatory properties on them [107]. These properties exhibit therapeutic potential for the treatment and prevention of numerous diseases. Brown algae, a significant source of marine polyphenols, have been historically utilized in food and traditional medicine by coastal communities to treat various ailments [108]. 

#### 4.3.1. Diphlorethohydroxycarmalol 

**Figure 4:**
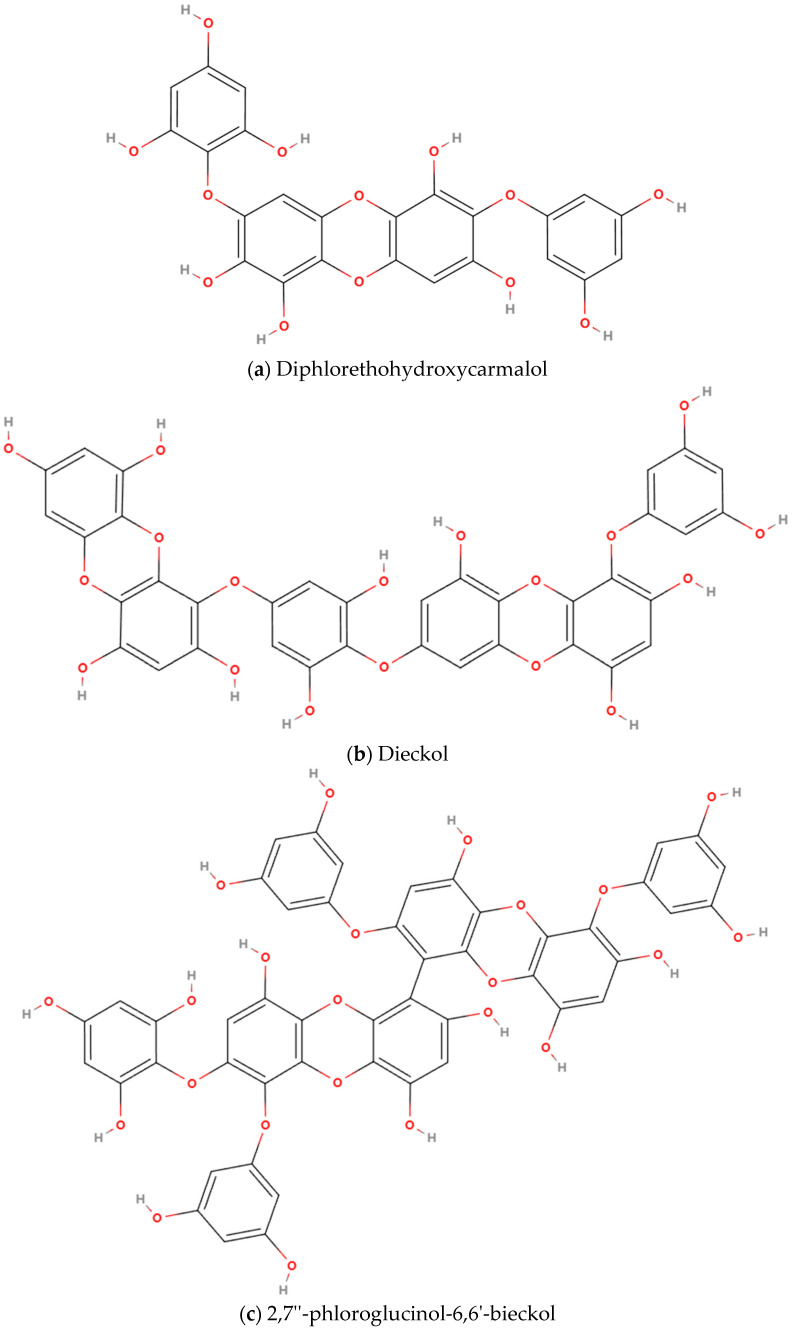
Chemical structures of polyphenols with potentially therapeutic effects to COPD skeletal muscle dysfunction: (**a**) Diphlorethohydroxycarmalol, (**b**) Dieckol, (**c**) 2,7″-phloroglucinol-6,6′-bieckol.

Diphlorethohydroxycarmalol (DPHC, 7-(3,5-dihydroxyphenoxy)-3-(2,4,6-trihydroxyphenoxy)dibenzo-p-dioxin-1,2,6,8-tetrol) is a polyphenolic compound extracted from *Ishige okamurae*, comprising four phloroglucinol units and functional -OH groups, with chemical formula C_24_H_16_O_13_ (Figure 4a) [109]. Studies have shown that DPHC can bind to TNF-α and inhibit the expression of pro-inflammatory factors in a dose-dependent manner [110]. Furthermore, DPHC ameliorates the abnormally elevated expression of muscle ring finger protein 1(MuRF-1) and muscle atrophy F-box (Atrogin-1) proteins induced by inflammation. A preclinical study systematically evaluated the effect of DPHC supplementation on Dex-induced skeletal muscle atrophy. The findings revealed that pre-treatment with *Ishige okamurae* extract or DPHC can significantly prevent the Dex-induced reduction in fatigue resistance and skeletal muscle atrophy [65]. Additionally, they suppressed the mRNA decrease of key factors triggered by Dex, including phosphoinositide 3-kinases (PI3K) and serine/threonine protein kinase B (Akt), both of which are associated with protein synthesis and muscle hypertrophy, as well as transient receptor potential vanilloid type 4 (TRPV4) and adenosine A1 receptor (A1R), which may be associated with the contractile function of skeletal muscle. Moreover, the study demonstrated that these treatments inhibited the mRNA expression of muscle-specific ubiquitin ligases, such as Atrogin-1 and MuRF-1. The aforementioned studies suggest that DPHC extracted from *Ishige okamurae* may enhance skeletal muscle function by exerting anti-inflammatory effects, promoting muscle protein synthesis and contraction capacity, and inhibiting muscle degradation (Table 4). These findings collectively position DPHC as a promising candidate for the treatment of skeletal muscle dysfunction in COPD.

#### 4.3.2. Dieckol and 2,7″-phloroglucinol-6,6′-bieckol

Dieckol (DK, 4-[4-[6-(3,5-dihydroxyphenoxy)-4,7,9-trihydroxydibenzo-p-dioxin-2-yl]oxy-3,5-dihydroxyphenoxy]dibenzo-p-dioxin-1,3,6,8-tetrol, C_36_H_22_O_18_; Figure 4b) and 2,7″-phloroglucinol-6,6′-bieckol (PHB, C_48_H_30_O_23_; Figure 4c), classified as phlorotannins, are primarily isolated from brown algae species, including *Eisenia bicyclis*, *Ecklonia cava*, and *Ecklonia stolonifera* [111]. Both compounds exhibit well-documented anti-inflammatory and antioxidant properties [112,113]. Kim et al. investigated the myogenic effects of polyphenolic phlorotannins derived from *Ecklonia cava*, demonstrating that DK and PHB significantly increased C2C12 myoblast proliferation and elevated creatine kinase activity, a biochemical marker of myogenic differentiation [66]. Mechanistic analyses revealed concurrent activation of IGF-1 signaling pathways and MyoD-mediated myogenesis, along with suppression of myostatin-driven proteolytic signaling. Molecular docking simulations further confirmed stable binding interactions between these phlorotannins and critical targets, including insulin-like growth factor-1 receptor (IGF-1R) and myostatin, key regulators of muscle metabolism. Emerging experimental evidence supports DK and PHB as promising therapeutic candidates for addressing COPD-related skeletal muscle dysfunction, mediated by their dual capacity to reduce oxidative stress and modulate protein degradation homeostasis (Table 4), highlighting its therapeutic potential.

#### 4.3.3. Miscellaneous Polyphenols

Phlorotannins, secondary polyphenolic metabolites uniquely biosynthesized in marine algae, are structurally composed of phloroglucinol (*1,3,5-trihydroxybenzene*) subunits polymerized through diverse linkage patterns. These compounds exhibit heterogeneous molecular architectures ranging from low-molecular-weight oligomers to high-molecular-weight polymers, with experimentally demonstrated antioxidant capabilities [114]. Kwon et al. [115] investigated the therapeutic potential of marine oligomeric polyphenols (MOPs), which are primarily composed of phlorotannins isolated from brown algae, in modulating body composition and physical performance among elderly sarcopenia patients. A 4-week intervention with MOPs supplementation significantly enhanced skeletal muscle mass, increased the percentage of FFMI, and improved balance function, concurrently reducing the completion time of the 2.4-m timed up-and-go test in older adults with sarcopenia. The observed improvements in sarcopenia-related musculoskeletal parameters and postural stability provide empirical evidence for marine-derived polyphenols in sarcopenia management (Table 4). These findings may further inform therapeutic strategies for COPD skeletal muscle dysfunction.

**Table 4:** Potential Ameliorative Effects of Polyphenols in COPD Skeletal Muscle Dysfunction.

Ref.	Object, Sample Size	Intervention	Origin	Administration Route	Dose	Duration	Primary Results
Ryu B (2022) [65]	ICR mice with skeletal muscle atrophy (10)	DPHC/*Ishige okamuraede* extract	*Ishige okamuraede*	Oral gavage	DPHC: 2.41 mg/kg/day *Ishige okamuraede* extract: 50 mg/kg/d; 100 mg/kg/d; 200 mg/kg/d	38 days	Grip strength ↑; Time of ladder climbing ↑; Lean mass of calf muscle ↑; Thickness of calf muscle↑; Gastrocnemius thickness ↑; CSA of gastrocnemius fiber ↑; Soleus muscle thickness ↑; Fiber diameter of soleus muscle ↑; MuRF-1 mRNA of gastrocnemius ↓; Atrogin-1 mRNA of gastrocnemius ↓; PI3K mRNA of gastrocnemius ↑; Akt mRNA of gastrocnemius ↑; TRPV4 mRNA of gastrocnemius ↑; A1R mRNA of gastrocnemius ↑; Myostatin mRNA of gastrocnemius ↑
Kim SY (2020) [110]	Inflammatory C2C12 myotube	DPHC	*Ishige okamuraede*	-	1.56 μg/mL; 3.125 μg/mL; 6.25 μg/mL; 12.5 μg/mL	1 h	MuRF-1 protein ↓; Atrogin-1 protein ↓
Kim, S.Y (2021) [66]	C2C12 myotube	DK	*Ecklonia cava*	-	5 nM; 10 nM; 20 nM	24 h	CK activity ↑; p-Smad2/3↓; Smad4 protein ↓; p-Akt protein ↑; p-FoxO protein ↑; MyoD ↑;
Kim, S.Y (2021) [66]	C2C12 myotube	PHB	*Ecklonia cava*	-	5 nM; 10 nM; 20 nM	24 h	CK activity ↑; p-Smad2/3 protein ↓; Smad4 protein ↓; p-Akt protein ↑; p-FoxO protein ↑; MyoD ↑
Kwon, I.S. (2021) [115]	Elderly Individuals with sarcopenia (10/10)	MOPs	Brown algae	Oral	One spoon (0.7 g) of Mannas™/d, Mannas™ with 1% MOPs	4 weeks	SSM ↑; %FFMI ↑; The 2.4 m up and go test ↓

Atrogin-1, muscle atrophy F-box; Akt, protein Kinase B; A1R, adenosine A1 receptor; CK, creatine kinase; DK, Dieckol; DPHC, diphlorethohydroxycarmalol; FoxO, forkhead box O; MyoD, myoblast determinant protein 1; MuRF-1, muscle ring finger protein 1; PI3K, phosphoinositide 3-kinases; TRPV4, transient receptor potential vanilloid type.

### 4.4. Peptides

Marine-derived bioactive peptides are widely distributed and exhibit characteristics such as low toxicity, multifunctionality, and high bioavailability [116]. As a result, they are widely explored in the research and development of drugs and functional foods. Due to their ease of decomposition and utilization by the human body, marine-derived bioactive peptides can serve as bioactive components for the formulation of functional foods. To date, no studies have specifically focused on the effects of marine peptides on muscle function and exercise capacity in COPD patients. However, preclinical studies in mice demonstrate that marine peptide supplementation significantly enhances anti-fatigue capacity, suggesting potential translational applications for managing COPD skeletal muscle dysfunction.

#### 4.4.1. Oyster Peptides

Oysters are nutritionally dense marine bivalves, providing an excellent source of bioactive compounds. Oyster-derived bioactive polypeptides exhibit significant anti-fatigue properties. Xiao et al. [67] demonstrated that 4-week supplementation with oyster-derived polypeptides significantly extended exhaustive swimming time in mice while reducing lactate and BUN accumulation. This treatment not only mitigated hepatic and muscular glycogen depletion but also enhanced gut microbiota diversity. Mechanistic investigations revealed upregulated mRNA expression of fatigue-related factors phosphoenolpyruvate carboxykinase (PEPCK) and AMP-activated protein kinase (AMPK), accompanied by increased antioxidant enzyme SOD and glutathione peroxidase (GSH-Px) activities as well as downregulated TNF-α mRNA expression. A subsequent study extended these findings by exploring the anti-fatigue effects of oyster polypeptides and their synergy with exercise [117]. Daily administration of 0.4 mg/g oyster polypeptides for 14 days replicated the previously observed improvements in swimming endurance, BUN reduction, and hepatic glycogen preservation. Notably, the combination of polypeptide supplementation and swimming exercise training synergistically enhanced both swimming performance and gastrocnemius glycogen content. Molecular analyses detected elevated HO-1 expression in gastrocnemius muscle, with combined intervention groups showing increased SOD, catalase (CAT), and GSH-Px activities. While oyster polypeptide supplementation upregulated energy metabolism regulators AMP-activated protein kinase (AMPK) and PGC-1α, the exercise–polypeptide combination specifically enhanced glucose transporter type 4 (GLUT4) expression. Molecular docking simulations validated stable binding interactions between oyster polypeptide sequences and both AMPK and HO-1. These findings collectively suggest that oyster polypeptides mitigate skeletal muscle fatigue through AMPK/HO-1-mediated enhancement of energy metabolism and antioxidant capacity, modulation of gut microbiota composition, and suppression of inflammatory pathways (Table 5). The observed synergy between polypeptide supplementation and exercise establishes a framework for developing combinatorial therapies to improve skeletal muscle function. The dual action on muscular fatigue resistance and gut microbial regulation positions oyster polypeptides as promising candidates for addressing COPD skeletal muscle dysfunction.

#### 4.4.2. Pyropia Yezoensis Peptides

*Pyropia yezoensis* is a species of red seaweed with an exceptionally high protein content by mass, exceeding that of traditional high-protein foods like soybeans [118]. It represents an abundant source of bioactive peptides. Peptides derived from *Pyropia yezoensis* have multiple bioactivities, including antioxidant, anti-tumor, and anti-inflammatory activities. Recent investigations demonstrated that a 24-h intervention with *Pyropia yezoensis* peptides (500 ng/mL) can effectively alleviate Dex-induced reduction in myotube diameter and increases in the mRNA and protein expression levels of MuRF-1 and Atrogin-1 [68]. Moreover, another study revealed that 24-h exposure to *Pyropia yezoensis* peptides suppressed Dex-induced activation of both the ubiquitin–proteasome system and autophagic-lysosomal pathway in C2C12 myotubes [119]. The intervention also counteracts the suppression of protein synthesis pathways mediated by IGF-1 and Akt/mammalian target of rapamycin (mTOR). Meanwhile, it reduces the total and nuclear protein expression levels of forkhead box o1 (FoxO1) and FoxO3a. In addition, the treatment increased the phosphorylation of FoxO1 and FoxO3a while attenuating the conversion of LC3-I to LC3-II, a hallmark of autophagosome formation. These findings suggest that *Pyropia yezoensis* peptides supplementation may alleviate Dex-induced myotube atrophy by modulating multiple pathways: inhibiting autophagy and oxidative stress, enhancing protein synthesis, and reducing protein degradation (Table 5), providing feasible evidence for the rehabilitation of COPD skeletal muscle dysfunction.

#### 4.4.3. Hippocampus Peptides

The seahorse/*hippocampus* (*Syngnathidae* family), a traditional Chinese medicinal resource, contains abundant proteins and essential amino acids in its tissue, establishing it as a valuable substrate for bioactive peptide production. An investigation evaluated the anti-fatigue properties of *hippocampus* polypeptides in murine models through daily oral administration at 0.15, 0.5, or 1.5 mg/g body weight for 4 consecutive weeks [69]. The mid- and high-dose regimens (0.5 and 1.5 mg/g) significantly prolonged exhaustive swimming duration while enhancing post-exercise blood glucose and hepatic glycogen levels. Concomitant reductions in blood lactate and serum BUN concentrations were observed, suggesting improved hepatic glycogen utilization or storage capacity to stabilize glycemic homeostasis and enhance exercise tolerance. In vitro antioxidant assays demonstrated that *hippocampus* peptides scavenged both 2,2-diphenyl-1-picrylhydrazyl (DPPH) radicals and hydroxyl radicals (•OH). These findings collectively demonstrate the capacity of *hippocampus* peptides to ameliorate exercise-induced fatigue through metabolic modulation and antioxidant activity (Table 5). The observed dose-dependent improvements in energy substrate management and fatigue biomarker modulation provide support for further exploration of *hippocampus* peptides as potential therapeutic agents for mitigating skeletal muscle dysfunction in COPD patients.

**Table 5:** Potential Ameliorative Effects of Peptides in COPD Skeletal Muscle Dysfunction.

Ref.	Object, Sample size	Intervention	Origin	Administration Route	Dose	Duration	Primary Results
Lin, S (2024) [117]	Kunming mice (10/10)	Oyster peptides	Crassostrea plicatula Gmelin	Gavage	0.4 mg/(g·d)	14 days	Swimming time ↑; Liver glycogen content ↑; BUN ↓; LDH ↓; lactic acid ↓; AMPK ↑; PGC-1α ↑; HO-1↑
Xiao, M. (2020) [67]	Kunming mice (20/20)	Oyster polypeptides	Oyster	Gavage	200 mg/kg/d; 400 mg/kg/d; 600 mg/kg/d	4 weeks	Swimming time ↑; lactic acid ↓; BUN ↓; SOD activity ↑; GSH-Px activity ↑; PEPCK mRNA ↑; AMPK mRNA ↑
Lee MK (2019) [119]	Atrophic C2C12 myotubes	*Pyropia yezoensis* peptide	*Pyropia yezoensis* Ueda	-	500 ng/mL	24 h	p-IGF-IR ↑; p-IRS-1 ↑; p-Akt ↑; p-mTOR ↑; Raptor protein ↑; p70S6K ↑; p-4EBP1 ↑; p-p70S6K ↑; p-S6 ↑; eIF4E ↑; FoxO3a ↓; FoxO1 ↓; p-FoxO3a ↑; p-FoxO1 ↑; conversion of LC3-I to LC3- II ↓; cathepsin-L ↓
Lee MK (2017) [68]	Atrophic C2C12 myotubes	*Pyropia yezoensis* peptide	Synthesis	-	500 ng/mL	24 h	Myotubes diameter ↑; MuRF-1 mRNA ↓; MuRF-1 protein ↓; Atrogin-1 mRNA ↓; Atrogin-1 protein ↓; 20S proteasome activity ↓
Guo, Z [69]	ICR mice (20/20)	*Hippocampus* peptides	*Hippocampus*	Gavage	0.15 mg/g/d; 0.5 mg/g/d; 1.5 mg/g/d	4 weeks	Exhausted swimming time ↑; Concentration of hepatic glycogen ↑; blood lactate ↓; BUN ↓

AMPK, AMP-activated protein kinase; Akt, protein Kinase B; Atrogin-1, muscle atrophy F-box; BUN, blood urea nitrogen; eIF4E, eukaryotic initiation factor 4E; FoxO, forkhead box O; HO-1, heme oxygenase-1; IGF-IR, insulin-like growth factor 1 receptor; IRS-1, insulin receptor substrate 1; GSH-Px, glutathione peroxidase; LDH, lactate dehydrogenase; mTOR, mammalian target of rapamycin; MuRF-1, muscle ring finger protein 1; PGC-1α, peroxisome proliferator-activated receptor gamma coactivator 1α; PEPCK, phosphoenolpyruvate carboxykinase; p70S6K, p70 ribosomal protein s6 kinase; SOD, superoxide dismutase; S6, S6 Kinase; 4EBP1, eukaryotic translation initiation factor 4e-binding protein.

### 4.5. Carotenoids

Approximately 750 naturally occurring carotenoids have been identified to date, with over 250 classified as marine-derived compounds [120]. Carotenoids are biosynthesized by diverse marine organisms including animals, algae, and microorganisms [121]. Marine carotenoids exhibit potent antioxidant, anti-inflammatory, and cytoprotective properties, positioning them as key bioactive agents for preventing oxidative stress-related pathologies. Structurally diverse representatives with documented bioactivities include β-carotene, fucoxanthin, peridinin, diatoxanthin, alloxanthin, and astaxanthin [121]. Epidemiological analyses have demonstrated that elevated serum carotenoid levels correlate with preserved walking speed and muscle strength in elderly populations [81,122,123], while showing inverse associations with COPD incidence [124,125]. These findings collectively underscore the dual benefits of carotenoids in maintaining musculoskeletal homeostasis and attenuating COPD pathogenesis. Investigations to date have revealed that specific carotenoids exert positive modulatory effects on both skeletal muscle architecture/function and pulmonary symptomatology in COPD, including astaxanthin, fucoxanthin, and β-carotene, β-cryptoxanthin, lutein, and zeaxanthin.

#### 4.5.1. Astaxanthin

**Figure 5:**
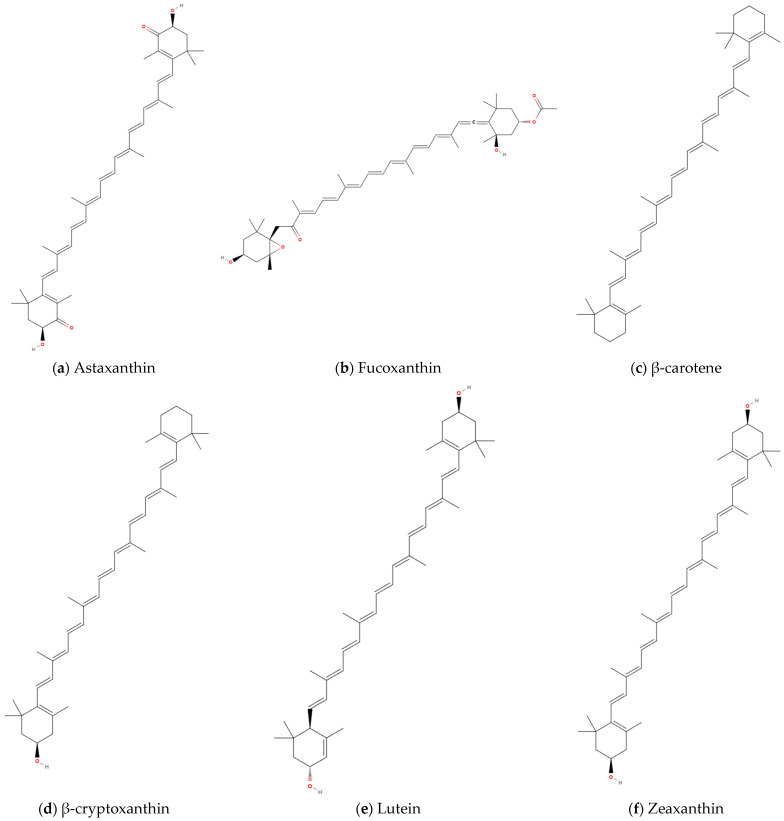
Chemical structures of carotenoids with potentially therapeutic effects on COPD skeletal muscle dysfunction: (**a**) Astaxanthin, (**b**) Fucoxanthin, (**c**) β-carotene, (**d**) β-cryptoxanthin, (**e**) Lutein, (**f**) Zeaxanthin.

Astaxanthin (AST; 3,3′-dihydroxy-β,β′-carotene-4,4′-dione; Figure 5a), a liposoluble marine xanthophyll carotenoid with the chemical formula C_40_H_52_O_4_, exhibits multifunctional biological activities. It is ubiquitously distributed across marine taxa, including microalgae, fungi, yeasts, crustaceans (e.g., shrimp and lobsters), and bacteria [126]. AST has been approved by Food and Drug Administration (FDA) for aquaculture applications and recognized as a Generally Recognized as Safe (GRAS) food additive, with subsequent authorization by the European Food Safety Authority (EFSA). According to the EFSA, the current acceptable daily intake of AST is established at 0.034 mg/kg BW, whereas the FDA permits its application at concentrations up to 12 mg per day [127]. Each ionone ring in the molecule contains both -OH and keto functional groups, enabling electron transfer to neutralize free radicals and form stabilized byproducts, effectively terminating radical-mediated chain reaction [128,129]. This unique molecular architecture makes AST exhibit strong antioxidant capacity [130].

Accumulating evidence has demonstrated the inhibitory effects of AST on skeletal muscle atrophy induced by diverse pathogenic mechanisms [70,71,72,73]. Mano et al. [70] reported that 12-week porcine pancreatic elastase (PPE) instillation significantly reduced the mass of the soleus muscle, the proportion of type I fibers, and the CSA of both type I and type IIa fibers. However, AST supplementation initiated at week 4 was effective in mitigating these pathological alterations. Meanwhile, AST downregulated the pathological overexpression of the Atrogin-1 protein. Yu et al. [71] administered 20-day dose-gradient AST supplementation to tumor cachexia mice and observed marked attenuation of muscle fiber CSA decline, along with reduced expression of muscle atrophy-related factors MuRF-1 and Atrogin-1. Mechanistically, AST restored mitochondrial dynamics homeostasis, suppressing fission mediators Drp1 and Fis1 while enhancing fusion regulator MFN2 expression. Additionally, it decreased mRNA expressions of autophagy-related factors PINK1, BNIP-3, and LC3B. Yue et al. [72] demonstrated that concurrent AST supplementation during Dex administration significantly inhibited Dex-induced declines in murine exercise endurance, forelimb grip strength, skeletal muscle mass, and CSA. Moreover, AST counteracted ubiquitin–proteasome activation while activating MyoD1, indicating dual regulation of protein degradation and synthesis pathways. Shibaguchi et al. [73] revealed that 14-day AST pretreatment prior to 10-day hindlimb immobilization prevented cast-induced muscle mass loss, increased SOD1 protein expression, suppressed cathepsin L, calpain, and ubiquitin overexpression, and maintained heat shock protein 72 (HSP72) expression levels.

The dual anti-inflammatory and antioxidant capacities of AST mediate protection against cigarette smoke (CS)-induced COPD pathogenesis [131]. Studies have demonstrated that AST alleviates oxidative stress, DNA damage, and apoptosis triggered by cigarette smoke extract (CSE) through suppression of NADPH oxidase-related signaling pathways, highlighting its therapeutic potential in pulmonary disorders [132]. In a murine model of CSE-induced emphysema, Deng et al. [131]. observed that concurrent AST supplementation significantly attenuated emphysematous changes, small airway fibrosis, oxidative stress, and inflammatory responses. Mechanistic investigations using in vitro experiments revealed that AST directly binds to SIRT1, promoting SIRT1-mediated deacetylation of Nrf2. This interaction enhances the release of antioxidant enzymes via Nrf2 activation while suppressing NF-κB p65 transcriptional activity to inhibit inflammatory cascades. Similarly, Kubo et al. [133] reported that AST supplementation inhibits CS exposure-induced pulmonary emphysema by activating the Nrf2/HO-1 signaling axis and reducing inflammatory cytokine levels in alveolar tissue.

These findings indicate that AST supplementation exerts a favorable effect on improving COPD and skeletal muscle dysfunction induced by multiple causes (Table 6). Existing evidence supports that AST potentially enhances skeletal muscle function in COPD by modulating oxidative stress, mitochondrial autophagy, and the equilibrium between mitochondrial fission and fusion.

#### 4.5.2. Fucoxanthin

Fucoxanthin (FX, C_42_H_58_O_6_; Figure 5b), a carotenoid derived from brown algae, features an allenic bond, a 5,6-monoepoxide group, and nine conjugated double bonds. This structural configuration enables its capacity to quench singlet oxygen and scavenge free radicals [134], conferring potent antioxidant properties associated with myoprotective effects [74,75,76]. In vitro studies demonstrated that FX attenuates Dex-induced reductions in C2C12 myotube diameter and myosin heavy chain (MHC) expression, while suppressing the upregulation of atrophy-related ubiquitin ligases Atrogin-1 and MuRF1 [74]. Further investigations revealed that FX exerts myoprotective effects via SIRT1-dependent pathways by mitigating protein degradation, enhancing mitochondrial biogenesis and function, and modulating apoptosis–autophagy crosstalk. Another study demonstrated that FX pretreatment effectively counteracts Dex-induced skeletal muscle atrophy and suppresses the elevation of lipid peroxidation marker MDA [75]. Concomitantly, FX inhibits AMPK phosphorylation while upregulating COX4 and mTOR protein expression. Fucoxanthinol (FXOH) is a metabolite of FX. Pretreatment with FXOH prevents hydrogen peroxide (H_2_O_2_)-induced reductions in C2C12 myotube CSA and suppresses ROS accumulation [76]. Additionally, FXOH reduces triacylglycerol (TG) content in 3T3-L1 adipocytes through enhanced glycerol and free fatty acid release, suppression of lipogenic factors, and induction of lipolysis-promoting factors [76]. FX and FXOH prevent skeletal muscle atrophy through antioxidant activity, mitochondrial function modulation, and homeostatic regulation of autophagy–apoptosis balance, as summarized in Table 6. These combined properties provide novel therapeutic insights for managing COPD patients with obesity-associated skeletal muscle dysfunction.

#### 4.5.3. β-Carotene

β-carotene (C_40_H_56_; Figure 5c), a naturally occurring orange pigment, serves as a well-established antioxidant and the primary dietary precursor of vitamin A and other fat-soluble vitamins [135]. Composed of eight isoprene units, β-carotene features a conjugated double-bond system flanked by β-ionone rings at both termini and interconnected by a polyene chain. This structure confers the ability to quench singlet oxygen and scavenge free radicals, thereby protecting cells against oxidative damage [136]. Emerging evidence demonstrates that β-carotene exerts antioxidant-mediated effects by inducing muscle hypertrophy, promoting muscle health, and counteracting multifactorial atrophy [77,78,79]. Ogawa et al. [78] revealed that β-carotene dose-dependently suppresses H_2_O_2_-induced C2C12 myotube atrophy and protein ubiquitination, while inhibiting FoxO3a nuclear translocation. Subsequent in vivo investigations showed that 2-week β-carotene supplementation (0.5 mg/day) attenuates muscle mass loss at 3 days post-denervation, concomitant with reduced levels of oxidative stress marker thiobarbituric acid reactive substances (TBARS) and protein ubiquitination. Notably, these protective effects were observed specifically in the soleus muscle rather than the gastrocnemius. Kitakaze et al. [79] further demonstrated that equivalent β-carotene supplementation (0.5 mg/day) for 2 weeks induces skeletal muscle hypertrophy, enhances muscle mass and peak contractile force, upregulates mRNA expression of protein synthesis regulators IGF-1 and IGF-Ea, while suppressing ubiquitin conjugate formation. Extending the supplementation period, Kim et al. [77] reported that 5-week β-carotene administration inhibits cancer cachexia by preventing skeletal muscle mass loss, suppressing lipolysis and adipose browning, mitigating systemic inflammation, and modulating gut microbiota composition and diversity. As a natural antioxidant, β-carotene exhibits rapid skeletal muscle protective effects following short-term supplementation (Table 6), suggesting potential therapeutic value for preventing disuse- or neuropathy-induced muscle atrophy, particularly in oxidative muscle fibers.

#### 4.5.4. β-Cryptoxanthin

β-Cryptoxanthin (C_40_H_56_O; Figure 5d), an oxygenated carotenoid, shares structural similarities with β-carotene. Its conjugated double bond system and polyene chain confer potent antioxidant capacity, while the additional -OH functional group enhances reactivity under specific oxidative conditions compared with β-carotene [137]. Predominantly derived from red algae in marine ecosystems [82]. It exhibits provitamin A activity and demonstrates superior anti-inflammatory, antioxidant, and anti-obesity effects compared to other carotenoids [138], particularly in modulating inflammatory biomarkers [139]. Clinical studies have identified a significant correlation between low serum β-cryptoxanthin levels and reduced lean muscle mass in aging populations [140]. Mechanistically, β-cryptoxanthin promotes skeletal muscle hypertrophy through autophagy regulation [78,80]. Experimental evidence shows its capacity to mitigate H_2_O_2_-induced downregulation of MHC and tropomyosin expression in C2C12 myotubes [78]. In accelerated aging mouse models, 15-week β-cryptoxanthin supplementation (0.5 mg/day) attenuated declines in grip strength, soleus muscle mass, and CSA of fiber, without significant effects on gastrocnemius, soleus, or plantaris muscles [80]. Concurrently, it suppressed age-related increases in autophagy markers including Bcl-2-interacting protein 1 (Beclin-1), p62, LC3-I, LC3-II, and ubiquitin conjugates, while reducing p62-positive fiber proportions. In vitro mechanistic studies confirmed that β-cryptoxanthin preferentially targets autophagic-lysosomal pathways over ubiquitin–proteasome systems. These findings underscore the therapeutic potential of β-cryptoxanthin in counteracting muscle atrophy associated with senescence and oxidative stress (Table 6), providing a mechanistic rationale for its application in COPD skeletal muscle dysfunction, particularly for enhancing oxidative muscle fiber structure and function.

#### 4.5.5. Lutein and Zeaxanthin

Lutein and zeaxanthin, structurally analogous carotenoids sharing the chemical formula C_40_H_56_O_2_, exhibit differential -OH group positioning that distinguishes their molecular configuration (Figure 5e, Figure 5f). Like other carotenoids, these marine-derived compounds primarily derived from algae exhibit potent antioxidant activity [82]. Accumulating evidence indicates their beneficial effects on skeletal muscle health [81,141,142,143]. Investigations reveal positive correlations between serum lutein/zeaxanthin levels and physical activity metrics [141], with higher dietary intake associated with attenuated age-related grip strength decline [81]. A 4-week intervention administering 3 capsules with lutein and zeaxanthin as well as 250 mL milk to elderly individuals with reduced physical activity significantly elevated plasma lutein and zeaxanthin concentrations. Elevated plasma levels of lutein positively correlated with enhanced physical activity duration and inversely correlated with sedentary behavior [142]. Complementary rodent studies demonstrated that 6-week supplementation with lutein-fortified milk increased voluntary running distance and gastrocnemius muscle mass in rats [143]. The demonstrated capacity of lutein and zeaxanthin to enhance skeletal muscle function positions these compounds as promising supplemental agents for managing COPD skeletal muscle dysfunction (Table 6).

**Table 6:** Potential Ameliorative Effects of Carotenoids in COPD Skeletal Muscle Dysfunction.

Ref.	Object, Sample Size	Intervention	Origin	Administration Route	Dose	Duration	Primary Results
Yue, H (2025) [72]	Skeletal muscle atrophy Male C57Bl/6J mice (8)	AST	*Haematococcus* *pluvialis*	Intraperitoneal injection	30 mg/kg/d, 60 mg/kg/d, 120 mg/kg/d	4 weeks	Body weight ↑; Time and distance exercise fatigue experiment ↑; Forelimb grip strength ↑; Hanging time ↑; Skeletal muscle mass ↑; CSA of myofibres ↑; MuRF-1 protein ↓; MyoD1 ↑
Yu X (2024) [71]	C26 tumor-bearing cancer cachexia male BALB/c mice (8)	AST	ND	ND	30 mg/kg/day, 60 mg/kg/day, 120 mg/kg/day	4 weeks	Body weight ↑; Tumor-free body weight ↑; Food intake ↑; Skeletal muscle mass ↑; skeletal muscle weight/tumor-free body weight ↑; CSA of myofibres ↑; Myosin heavy chain ↑; MuRF-1 mRNA ↓; MuRF-1 protein ↓; Atrogin-1mRNA ↓; Atrogin-1 protein ↓
Mano Y (2022) [70]	C57BL/6J COPD mice (10)	AST	ND	Feed the CE-2 diet containing AST	0.02%	8 weeks	Proportion of type I fibers ↑; CSA of type I fibers ↑; CSA of type IIA fibers ↑; Atrogin-1protein ↓
Shibaguchi, T. (2016) [73]	Wistar rat with atrophy	AST	*Haematococcus pluvialis*	Feed the CE-2 diet containing AST	0.04%, 0.2%	10 days	Degree of atrophy↓; SOD1 protein ↑; Cathepsin L ↓; Ubiquitin ↓
Deng, M. (2023) [131]	C57BL/6J mice with COPD (12/12)/Human bronchial epithelial cells with CSE	AST	Commercial sources	Ganage	10 mg/kg/d, 50 mg/kg/d, and 100 mg/kg/d/ 50 μM, 100 μM	29 days/24 h	MLI ↓; MAA ↓; Body weight ↑; MMP-9 ↓; TIMP-1 ↑; Airway inflammation ↓; Peribronchiolar collagen deposition ↓; E-cadherin ↑; α-SMA ↓ SIRT1 protein ↑/MDA ↓; T-AOC ↑; SOD ↑; GSH ↑; Nrf2 protein↑; HO-1 protein↑; TNF-α↓; IL-6 ↓; p-NF-κBp65 protein ↓; SIRT1 protein ↑
Zhiyin, L. (2021) [74]	C2C12 myotube with atrophy	FX	Commercial sources	-	10 μM	24 h	Atrogin-1 protein ↓; MuRF1 protein ↓; Diameter of myotube ↑; MHC protein ↑ ATP production ↑; SIRT1 protein ↑; p-Akt/Akt ↑; FoxO3a protein ↑; p-FoxO3a/FoxO3a ↑; AC-FoxO3a ↑; PGC-1αprotein ↑; Nrf1 protein ↑; TFAM protein ↑; Bax/Bcl-2 ↓; Cleaved caspase-3 protein ↓; Apoptosis cells ↓
Yoshikawa, M. (2021) [75]	ICR mice with atrophy (7/8)	FX	Commercial sources	Feed a mixture containing 0.2% Fx	0.2%	27 days	TA weight ↑; MDA ↑; p-AMPK/AMPK ↓; COX4 protein ↑; p-mTOR/Mtor ↑
Yoshikawa, M. (2020) [76]	C2C12 myotube with H_2_O_2_/3T3-L1 adipocytes	FXOH	Commercial sources	-	5 μM/2.5 μM, 5 μM and 10 μM	24 h/72 h	Area of myotubes ↑; MYC protein ↑; ROS ↓;/TG ↓; Glycerol release ↑; Fatty acid release ↑; lipolysis-associated proteins (ATGL, p-HSL, Perilipin and CGI-58) ↓; p-AMPK/AMPK ↑; p-ACC/ACC ↑; FAS ↓
Kim, Y. (2023) [77]	BALB/c with cancer cachexia	β-carotene	Commercial sources	Oral	0.5 mg/kg 2 mg/kg 2 t/w	5 weeks	Muscle mass ↑; Fat weight ↑; Adipocytes size ↑; lipolysis markers (ATGL and HSL) mRNA ↓; Brown adipocyte-specific genes (UCP1, PDK4, PGC-1α) mRNA ↓; Serum of IL-6 ↓; Serum of TNF-α ↓; Altered fecal microbiota structure; Intestinal flora diversity ↑
Kitakaze, T. (2015) [79]	Kwl:ddY mice (5/5)	β-carotene	Commercial sources	Gavage	0.5 mg/day	2 weeks	Muscle mass ↑; MVC ↑; IGF-1 mRNA ↑; IGF-Ea mRNA ↑
Ogawa, M. (2013) [78]	C2C12 myotube with H_2_O_2_/Denervated Kwl:ddY mice (15/15)	β-carotene	Commercial sources	-/Gavage	10 μM/0.5 mg/day	12 h/2 weeks	MHC ↑; Tropomyosin ↑; Atrogin-1 mRNA ↓; MuRF1 mRNA ↓; USP14 mRNA ↓; USP19 mRNA ↓; nuclear localization of FoxO3a ↓/Related soleus muscle mass ↑; TBARS ↓; ubiquitin conjugates ↓; Atrogin-1 mRNA ↓; MuRF1 mRNA ↓; USP14 mRNA ↓; USP19 mRNA ↓
Noguchi, M. (2020) [80]	SAMP1 mice (6~8/6~8)	β-cryptoxanthin	*Satsuma mandarin*	Gavage	0.5 mg/day	15 weeks	Grip Strength ↑; EDL muscle mass/body weight ↑; CSA of EDL muscle fiber ↑; MHC I ↑; Autophagy-related factors (Beclin-1, p62, LC3-I, and LC3-II) protein ↓; p62-positive Fiber ↓; Ubiquitin conjugates ↓
Ogawa, M. (2013) [78]	C2C12 myotube with H_2_O_2_	β-cryptoxanthin	ND	-	10 μM	12 h	MHC ↑; Tropomyosin ↑
Thomson, R.L. (2014) [142]	Older adults (20/19)	Lutein and Zeaxanthin	ND	Oral	Lutein: 21 mg/day; Zeaxanthin: 0.9 mg/day	4 weeks	Plasma lutein ↑; Plasma zeaxanthin ↑; Plasma lutein is negatively associated with sedentary time; Plasma lutein is positively associated with daily activity counts

AST, astaxanthin; Atrogin-1, muscle atrophy F-box; Akt, protein Kinase B; AC-FoxO3a, acetyl-forkhead box o3a; ATGL, adipose triglyceride lipase; ACC, acetyl-CoA carboxylase; AMPK, AMP-activated protein kinase; Bax, BCL2-associated x protein; Bcl-2, B-cell lymphoma-2; Beclin-1, Bcl-2-interacting protein 1; COX4, cytochrome c oxidase subunit 4; CGI-58, comparative gene identification-58; CSA, cross-sectional area; EDL, Extensor digitorum longus; FoxO3a, forkhead box O3a; FX, Fucoxanthin; FXOH, Fucoxanthinol; FAS, fatty acid synthase; GSH, glutathione; IL-6, interleukin 6; HSL, hormone-sensitive lipase; HO-1, heme oxygenase-1; IGF-Ea, insulin-like growth factor Ea; MuRF-1, muscle ring finger protein 1; MHC, myosin heavy chain; MDA, malondialdehyde; mTOR, mammalian target of rapamycin; MyoD1, myogenic differentiation 1; MVC, maximal voluntary contraction; MLI, mean linear intercept; MAA, mean alveolar area; MMP-9, matrix metalloproteinase-9; NF-κB, nuclear factor kappa-light-chain-enhancer of activated B cells; LC3, microtubule-associated protein 1A/1B-light chain 3; Nrf, nuclear respiratory factor; PGC-1α, peroxisome proliferator-activated receptor gamma coactivator 1α; PDK4, pyruvate dehydrogenase kinase 4; p62, sequestosome-1; SIRT1, Sirtuin 1; SOD, superoxide dismutase; TFAM, mitochondrial transcription factor A; TA, tibialis anterior; TG, triacylglycerol; T-AOC, total antioxidant capacity; TIMP, tissue inhibitor of metalloproteinases-1; TNF-α, tumor necrosis factor-alpha; UCP1, uncoupling protein 1; USP, ubiquitin specific peptidase; α-SMA, alpha-smooth muscle actin.

### 4.6. Combinatorial Antioxidants

The subcellular localization of antioxidants determines their capacity to scavenge specific free radical species based on physicochemical properties [144]. Synergistic interactions among antioxidants further enhance their bioavailability and cumulative efficacy through reciprocal absorption potentiation [145]. Furthermore, combinatorial antioxidant administration optimizes radical neutralization across cellular compartments while mitigating the pro-oxidant conversion risks inherent to high-dose monotherapy under specific physiological conditions [145]. Kawamura et al. [144] established a murine model of atrophy through unilateral hindlimb immobilization for 3 weeks, followed by a 2-week recovery period after cast removal. Dietary supplementation with astaxanthin, β-carotene, and resveratrol during both atrophy induction and recovery phases elicited effects on skeletal muscle regeneration. While single antioxidants normalized atrophic and contralateral muscle mass ratios, the triple combination promoted significant hypertrophy in previously immobilized limbs. Mechanistic analyses revealed selective upregulation of protein synthesis markers, including increased phosphorylation levels of mTOR and p70 ribosomal protein s6 kinase (p70S6K), and reduced protein carbonylation exclusively in the combinatorial treatment group. Subsequent work by Kawamura et al. [146] extended these findings to resistance training adaptations. The antioxidant triad enhanced exercise-induced improvements in maximal voluntary contraction (MVC) and fatigue resistance, concomitant with elevated resting oxygen consumption and reduced respiratory quotient. This low-dose combination synergistically promoted both myofiber hypertrophy and metabolic adaptations while circumventing the paradoxical oxidative stress associated with high-dose single-agent protocols [147]. Collectively, these advantages position combination antioxidant therapy as a viable strategy for preventing and managing muscle dysfunction (Table 7). Specifically, its dual capacity to synergistically enhance therapeutic efficacy while mitigating dose-dependent oxidative risks provides novel insights into addressing COPD skeletal muscle dysfunction.

### 4.7. Miscellaneous

#### 4.7.1. Gloiopeltis Tenax

*Gloiopeltis tenax,* an annual red alga widely distributed in intertidal and subtidal zones along continental coastlines, has demonstrated antioxidant properties in previous studies [148]. Kim et al. [147] investigated its therapeutic potential in an in vitro C2C12 myotube model, revealing that pretreatment with *Gloiopeltis tenax* extract attenuated Dex-induced myotube atrophy. This protective effect was mediated through activation of the Akt/mTOR signaling pathway, evidenced by elevated phosphorylation levels of Akt and mTOR, alongside the upregulated mRNA expression of PGC-1α and increased mtDNA/nuclear DNA (nDNA) ratios. In vivo validation studies demonstrated that intraperitoneal Dex administration in mice over 10 days induced skeletal muscle atrophy, impaired exercise performance, and reduced mtDNA content coupled with decreased expression of oxidative phosphorylation (OXPHOS) complex proteins. Notably, prophylactic supplementation with *Gloiopeltis tenax* extract for 7 days prior to Dex exposure effectively prevented these pathological alterations.

**Table 7:** Potential Ameliorative Effects of Combined Astaxanthin, β-Carotene, and Resveratrol in COPD Skeletal Muscle Dysfunction.

Ref.	Object, Sample Size	Intervention	Origin	Administration Route	Dose	Duration	Primary Results
Kawamura, A. (2020) [144]	ICR mice with atrophy	Astaxanthin, β-carotene, and resveratrol	Commercial sources	Oral	0.06% (*w*/*w*)	5 weeks	Muscle mass ↑; p-mTOR protein ↑; p-p70S6K protein ↑; carbonylation ↓
Kawamura, A. (2021) [146]	Healthy men (13/13)	Astaxanthin, β-carotene, and resveratrol	Salmon flakes, green and yellow vegetable juice, and lingonberry jam	Oral	ND	10 weeks	MVC ↑; oxygen consumption ↑; respiratory quotient ↓

MVC, maximal voluntary contraction; mTOR, mammalian target of rapamycin; p70S6K, p70 ribosomal protein s6 kinase.

#### 4.7.2. Undaria Pinnatifida

*Undaria pinnatifida* (a brown alga) harbors structurally distinct bioactive compounds, including polysaccharides, polyphenols, and carotenoids. Experimental evidence demonstrated that 8-week supplementation with *Undaria pinnatifida* extract enhanced exercise endurance, muscle mass, and type I myofiber proportion in murine models [149]. Mechanistic investigations revealed that the extract upregulated the expression of factors governing oxidative metabolism, fatty acid uptake, glucose transport, angiogenesis, and mitochondrial biogenesis, concomitant with activation of the estrogen-related receptor (ERR) and SIRT1-AMPK-PGC-1α signaling network. Subsequent phytochemical analysis identified fucoxanthin, hesperetin, and caffeic acid as key bioactive constituents within the extract capable of activating the aforementioned pathways. These data suggest that fucoxanthin, hesperetin, and caffeic acid in *Undaria pinnatifida* may coordinately modulate the ERR and SIRT1-AMPK-PGC-1α signaling axes to promote mitochondrial biogenesis, type I myofiber remodeling, and angiogenesis, thereby enhancing skeletal muscle functional capacity.

#### 4.7.3. Codium Fragile

*Codium fragile* (CF), a green alga of the Codiaceae family, harbors significant bioactive potential. A 10-week supplementation with CF extract markedly enhanced exercise endurance and muscle mass in murine models, as evidenced by improved running speed, distance, and total exercise duration [150]. Mechanistically, CF extract activated the Akt-mTORC1 pathway, upregulating protein expression of total myosin heavy chain (T-MHC), and phosphorylation levels of pro-myoprotein synthesis factors Akt, mTOR, p70S6K1, and eukaryotic translation initiation factor 4e-binding protein 1 (4EBP1). Meanwhile, it enhanced myogenic differentiation factors Myf5 and MyoD, along with mitochondrial regulators PGC-1α and SIRT1. In vitro experiments confirmed that the beneficial effects of CF on skeletal muscle are SIRT1-dependent. UPLC-QTOF-MS/MS analysis identified key bioactive constituents in CF, including lysophosphatidylcholine, canthaxanthin, retinoic acid, α-tocopherol, and unsaturated fatty acids, which contribute to its skeletal muscle-enhancing properties. These findings suggest that CF modulates the Akt-mTORC-SIRT1 axis to promote myogenesis and mitochondrial adaptation, with bioactive compounds acting synergistically to improve skeletal muscle function.

#### 4.7.4. Oysters

Oysters are an important component of marine resources, and some oyster hydrolysates have exhibited antioxidant capacity by scavenging free radicals. Intervention with fermented oyster extract for 4 weeks before Dex injection significantly reduced the Dex-induced decreases in gastrocnemius thickness and weight, CSA of muscle fiber, and grip strength [151]. Additionally, fermented oyster extract inhibited the mRNA upregulation of muscle atrophy-related ubiquitin ligases Atrogin-1 and MuRF-1. Moreover, fermented oyster extract reduced the expression of pro-inflammatory factors IL-6 and TNF-α in the gastrocnemius caused by Dex. It also suppressed the increase in NF-κB mRNA expression in the nucleus. Meanwhile, it mitigated the Dex-induced decrease in mRNA expression of antioxidant factors, including Nrf2 and HO-1. Fermented oyster extract also restored SOD activity and glutathione levels, which are key indicators of antioxidant defense. Furthermore, it inhibited the Dex-induced increase in kelch-like ECH-associated protein 1 (Keap1) mRNA expression and the NADP+/NADPH ratio. In summary, pre-treatment with fermented oyster extract can enhance cellular resilience against oxidative stress by the Keap1/Nrf2 axis and inflammation, highlighting its potential therapeutic application. 

**Figure 6:**
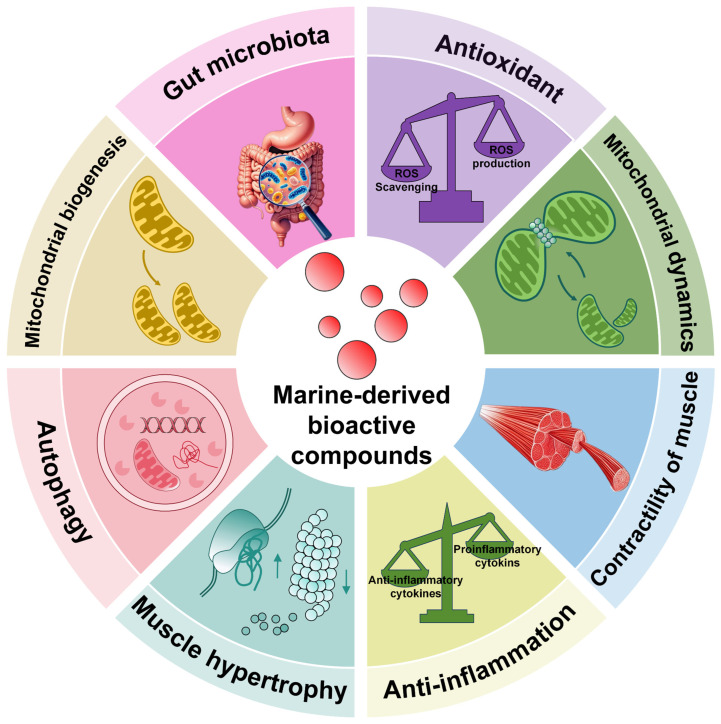
Bioactivities of marine-derived bioactive compounds in relation to COPD skeletal muscle dysfunction.

Extracts from *Gloiopeltis tenax*, *Undaria pinnatifida*, CF, and oysters exhibit therapeutic effects on skeletal muscle homeostasis through coordinated mechanisms involving antioxidant, anti-inflammatory, and mitochondrial-protective activities of marine-derived bioactive compounds (Table 8). These findings substantially strengthen existing evidence supporting the health benefits of supplementation of marine origin on skeletal muscle systems while highlighting promising therapeutic applications for ameliorating COPD skeletal muscle dysfunction through intervention with marine-derived bioactive compounds. The observed biological effects are attributed to the coordinated modulation of oxidative stress pathways, inflammatory cascades, and mitochondrial bioenergetics, consistent with established mechanisms underlying skeletal muscle homeostasis. The bioactivities of marine-derived bioactive compounds in relation to COPD skeletal muscle dysfunction are summarized in Figure 6.

## 5. Therapeutic Opportunities and Challenges of Marine-Derived Bioactive Compounds in COPD Skeletal Muscle Dysfunction

COPD is a multisystem disorder characterized by systemic manifestations, with skeletal muscle dysfunction emerging as a prevalent secondary extrapulmonary complication. This condition is clinically associated with impaired exercise performance, accelerated fatigue, and increased fracture risk in COPD patients [5]. Current therapeutic strategies, including pulmonary rehabilitation, nutritional supplementation, and neuromuscular electrical stimulation, have demonstrated efficacy in mitigating disease progression [12]. However, substantial challenges remain in achieving complete prevention or reversal of skeletal muscle dysfunction in advanced COPD. The established pathogenic roles of oxidative stress and chronic inflammation in COPD skeletal muscle dysfunction highlight the potential of antioxidant and anti-inflammatory agents as novel therapeutic candidates. This pathophysiological understanding creates significant opportunities for marine-derived bioactive compounds.

Accumulating evidence demonstrates that diverse marine-derived bioactive compounds exert multi-target beneficial effects on skeletal muscle health. This polypharmacological profile enhances therapeutic efficacy while improving safety margins through reduced tolerance development compared to single-target agents [154]. The inherent safety of these compounds is further supported by their natural origins in edible marine organisms, many of which have established applications in the food and cosmetic industries [155]. Notably, the synergistic benefits observed between marine bioactive supplementation and exercise regimens [92,96,97,117] provide a strategic framework for developing comprehensive and personalized COPD management protocols. Furthermore, preclinical evidence highlighting enhanced efficacy through antioxidant combination therapies [144,146] suggests innovative paradigms for marine compound applications. Such combinatorial approaches may effectively circumvent challenges associated with single therapeutic approaches, including tolerance development and dose-dependent toxicity observed with high-concentration single-compound regimens.

The primary limitation in developing marine-derived bioactive compounds for COPD skeletal muscle dysfunction is the scarcity of clinical validation. Most reported myoprotective and anti-atrophic effects originate from rodent models or in vitro experiments. This translational bottleneck likely stems from insufficient interdisciplinary collaboration, as we identified that the majority of researchers proposing skeletal muscle therapeutic potentials for marine-derived bioactive compounds lack clinical expertise, thereby significantly impeding their progression through clinical translation pipelines. A critical safety concern involves the dose-dependent duality of these compounds. Although marine bioactives generally exhibit favorable safety profiles at physiological concentrations, emerging evidence identifies toxicological risks at elevated doses, including organ-specific cytotoxicity and metabolic disruptions [147,156]. This dual-phase behavior, therapeutic at low doses versus toxic at high doses, necessitates rigorous dose optimization. However, the structural complexity inherent to marine-derived compounds complicates pharmacokinetic predictability, particularly regarding bioavailability, tissue-specific accumulation, and clearance mechanisms, posing significant challenges for establishing safe therapeutic windows [157]. Therefore, determining safe therapeutic dosage ranges represents a critical prerequisite for advancing marine bioactive compounds toward clinical translation. Bioavailability limitations further constrain clinical applicability. For instance, carotenoids’ lipid solubility enables broad tissue distribution, including central nervous system penetration, but results in inefficient intestinal absorption compared to hydrophilic antioxidants [158]. Current formulation innovations, such as lipid-based nanoencapsulation or co-administration with emulsifiers, show potential to enhance bioavailability but require systematic validation. Nevertheless, this will not present an insurmountable challenge in the long run. The stability and extraction of bioactive compounds also pose an unavoidable bottleneck. Phlorotannins, despite their documented capacity to mitigate muscle atrophy, exhibit extraction yields below 7% from natural sources like *Sargassum fusiforme* [159]. Nevertheless, progress in green extraction technologies (e.g., subcritical water extraction) and synthetic biology approaches for compound stabilization may address these production challenges, enabling standardized bioactive preparation.

Marine resources constitute a vast repository of bioactive compounds with substantial therapeutic potential. The exploration of marine-derived bioactive compounds emerges as a promising strategy for the prevention and management of COPD skeletal muscle dysfunction. To advance the development and translational progress of marine-derived bioactive compounds, concerted efforts are required to optimize purification techniques and stability enhancement protocols, foster interdisciplinary collaboration to facilitate clinical translation, and conduct systematic investigations into their toxicological profiles, safety parameters, and underlying molecular mechanisms. While these marine-derived bioactive compounds present significant opportunities for addressing COPD skeletal muscle dysfunction, their clinical implementation concurrently poses challenges related to bioactivity standardization and pharmacological validation.

## 6. Conclusions

This article briefly outlines the pathogenesis of COPD skeletal muscle dysfunction and focuses on marine-derived bioactive compounds with therapeutic potential for this condition. Current evidence indicates that various marine-derived bioactive compounds, including both purified and crude extracts from marine plants and animals, exhibit skeletal muscle health-promoting effects. Although most studies remain in the preliminary exploratory stage, several marine-derived bioactive compounds have entered clinical-phase investigations and demonstrated significant potential in promoting skeletal muscle health, including fucoidan, n-3 LC-PUFAs, MOPs, AST, lutein, and zeaxanthin. Notably, n-3 LC-PUFAs, AST, and MOPs exhibit additional anti-atrophic properties. Among these, n-3 LC-PUFAs are the only marine-derived bioactive compounds extensively studied in clinical settings for both skeletal muscle health enhancement and COPD skeletal muscle dysfunction management. However, their optimal effective and safe supplementation dosages require further exploration. Current studies report supplementation durations ranging from 12 days to 6 months, highlighting the necessity for long-term follow-up studies to comprehensively evaluate safety profiles. Mechanistic investigations in existing studies remain at an early stage, with more observational data than detailed confirmation of causal relationships. The combination of marine-derived bioactive compounds represents a promising strategy for future supplementation, potentially improving efficacy while mitigating drug resistance and toxicity. Moving forward, multidisciplinary collaborations are urgently needed to accelerate clinical translation in COPD populations, refine dosing regimens, and elucidate underlying mechanisms. Integrating these compounds with other therapeutic interventions may offer novel, safe, and effective approaches for the prevention and rehabilitation of COPD skeletal muscle dysfunction.

## Figures and Tables

**Table 1:** Potential Bioactivity of Marine-Derived Bioactive Compounds in COPD Skeletal Muscle Dysfunction.

Family	Compound	Origin	Bioactivity	Ref.
Polysaccharides	Fucoidan	*Brown algae* and *Undaria pinnatifida*	Anti-fatigue; Promote muscle synthesis; Enhance the contractility of muscle fibers; Enhance mitochondrial biogenesis; Promote the formation of an inflammatory environment after high-intensity exercise	[51,52,53,54]
	Chitosan	Chitin produced from the exoskeletons of arthropods	Anti-fatigue; Antioxidant; Promote glucose uptake, mitochondrial biogenesis; Alter mitochondrial respiratory chain complex	[55,56,57,58]
	Ulvan	Green algae	Antioxidant; Anti-inflammation	[59,60]
Lipids	DHA	Fish oil	Promote muscle synthesis; Anti-inflammation	[61,62,63]
	EPA	Fish oil	Promote muscle synthesis; Anti-inflammation	[61,62,63]
	FST	Brown algae	Antioxidant; Anti-inflammation;	[64]
Polyphenols	DPHC	*Ishige okamurae*	Promote muscle synthesis; Promote contraction capacity	[65]
	DK	*Ecklonia cava*	Promote muscle synthesis; Inhibit muscle degradation;	[66]
	PHB	*Ecklonia cava*	Promote muscle synthesis; Inhibit muscle degradation;	[66]
Peptides	Oyster peptides	Oyster	Antioxidant; Anti-fatigue; Anti-inflammation; Enhance energy metabolism; Modulate gut microbiota composition	[67]
	*Pyropia yezoensis* peptides	*Pyropia yezoensis*	Inhibit skeletal muscle atrophy; Inhibit autophagy; Promote muscle protein synthesis; Inhibit muscle protein degradation	[68]
	*Hippocampus* Peptides	*Hippocampus*	Anti-fatigue; Regulate metabolism; Antioxidant;	[69]
Carotenoids	AST	*Haematococcus* *pluvialis*	Antioxidant; Anti-inflammation; Promote muscle hypertrophy; Inhibit protein degradation; Regulate mitochondrial fission and fusion	[70,71,72,73]
	FX	Brown algae	Reduced protein degradation; Improves mitochondrial count and function; Apoptosis and autophagy; Antioxidant; Promote lipolysis; Inhibit lipogenesis	[74,75,76]
	β-carotene	Marine microorganisms and algae	Antioxidant; Anti-inflammation; Promote skeletal muscle hypertrophy; Inhibit skeletal muscle atrophy, lipolysis and fat browning; Modulate gut microbiota and diversity	[77,78,79]
	β-cryptoxanthin		Promote muscle synthesis; Inhibit autophagy	[78,80]
	Lutein	Algae	Antioxidant; Promote muscle synthesis;	[81]
	Zeaxanthin	Algae	Antioxidant; Promote muscle synthesis;	[81,82]

AST, astaxanthin; CF, DHA, docosahexaenoic acid; DK, Dieckol; DPHC, Diphlorethohydroxycarmalol; EPA, eicosapentaenoic acid; FX, Fucoxanthin; FST, Fucosterol; PHB, 2,7″-phloroglucinol-6,6′-bieckol.

**Table 8:** Potential Ameliorative Effects of Marine-Derived Miscellaneous in COPD Skeletal Muscle Dysfunction.

Ref.	Object, Sample Size	Intervention	Origin	Administration Route	Dose	Duration	Primary Results
Kim, S H (2024) [152]	C57Bl/6 mice with skeletal muscle atrophy	*Gloiopeltis tenax* extract	*Gloiopeltis tenax*	Oral	8 mg/kg/d	7 days	Myotube size ↑; Isometric strength of forelimb muscles ↑; Aerobic endurance ↑ Weight of the EDL ↑; Diameter of muscle fibers ↑; Atogin-1 mRNA ↓; MuRF-1 mRNA ↓
Ahn, J. (2020) [149]	C57BL/6 mice (10/10)	*Undaria pinnatifida* extract	Commercial sources	Add to the diet	0.25%	8 weeks	Running distance ↑; Maximum speed ↑ Total running time ↑; EDL weight ↑ Gastrocnemius weight ↑; CSA of gastrocnemius ↑; MHC1 protein ↑; MEF2 mRNA ↑; MEF2C mRNA ↑; Cyt C mRNA ↑; COX5a mRNA↑; Fatty acid uptake-related genes (FATP1, Apoe, Fabp4) ↑; Fatty acid oxidation-related genes (Acadm, PDK4, UCP3, CPT1a, CPT1b, and CPT2) ↑; Glucose uptake-related genes (GULT3, GULT4) ↑; CD31 intensity ↑; Angiogenesis markers genes (VEGFa, VEGFb, FGF1, ANGPT1, and ANGPT2) ↑; Mitochondria area ↑; Mitochondrial OXPHOS respiratory complex-related genes (NDUFS8, UQCRC1, ATP5a, SDHb, and COX5b) ↑; Complex I, III-V protein ↑; Nrf2 mRNA ↑; TFAM mRNA ↑; Nrf2 protein ↑; ERRγ protein ↑; ERRα protein ↑; SIRT1 protein ↑
Li, Y (2021) [153]	ICR mice with muscle atrophy	Oyster extracts	Crassostrea gigas	Oral	50 mg/kg/d, 100 mg/kg/d, 200 mg/kg/d	4 weeks	Body weight ↑; Muscle thickness of gastrocnemius ↑; Weight of gastrocnemius CSA of gastrocnemius fibers ↑; Strength of gastrocnemius ↑
Ahn, J. (2021) [150]	Old C57BL/6 mice	CF extracts	Commercial sources	AIN-93 M diet with CF	0.1% (*w*/*w*)	10 weeks	Running time ↑; Maximum speed ↑; Total running time ↑; Quadriceps weight, ↑; Soleus muscle ↑; CSA of soleus muscle fiber ↑; Skeletal muscle growth-related protein of quadriceps (T-MHC, p-AKT, p-mTOR, p70-S6K1, and p-4EBP1) ↑; Myf5 protein ↑; MyoD protein ↑ MHC1 protein ↑; MHC2a protein ↑; Complex I-V ↑; MEF2A ↑; MEF2C ↑; ERRγ protein ↑; PPARδ protein ↑; PGC-1α protein ↑; SIRT1 protein ↑; ERRαprotein ↑; Nrf1 protein ↑; Myoglobin protein ↑

Atrogin-1, muscle atrophy F-box; Acadm, acyl-CoA; dehydrogenase medium chain; ANGPT, angiopoietin; Apoe, apolipoprotein E; Akt, protein Kinase B; CD31, cluster of differentiation 31; COX5a, cytochrome c oxidase subunit 5a; CPT, carnitine palmitoyltransferase; Cyt C, cytochrome c; CSA, cross-sectional area; CF, Codium fragile; ERR, estrogen-related receptor; EDL, Extensor digitorum longus; Fabp, fatty acid binding protein; FATP1, fatty acid transport protein 1; FGF1, fibroblast growth factor; GLUT, glucose transporter; MEF, myocyte enhancer factor; MHC, myosin heavy chain; MyoD, myogenic differentiation 1; Myf5, myogenic factor 5; mTOR, mammalian target of rapamycin; MuRF-1, muscle ring finger protein 1; Nrf, nuclear respiratory factor; NDUFS8, NADH:ubiquinone oxidoreductase core subunit S8; OXPHOS, oxidative phosphorylation; PDK4, pyruvate dehydrogenase kinase 4; p70S6K, p70 ribosomal protein s6 kinase; PGC-1α, peroxisome proliferator-activated receptor gamma coactivator 1α; PPAR, peroxisome proliferator-activated receptor; SIRT1, sirtuin 1; SDH, succinate dehydrogenase; TFAM, mitochondrial transcription factor A; T-MHC, total myosin heavy chain; UCP3, uncoupling protein 3; UQCRC1, ubiquinol–cytochrome c reductase core protein 1, VEGF, vascular endothelial growth factor; 4EBP1, eukaryotic translation initiation factor 4e-binding protein 1.

## Data Availability

Not applicable.
